# Bacterial community diversity and potential eco-physiological roles in toxigenic blooms composed of *Microcystis*, *Aphanizomenon* or *Planktothrix*

**DOI:** 10.3389/fmicb.2025.1655370

**Published:** 2025-12-16

**Authors:** Joanna Mankiewicz-Boczek, Arnoldo Font-Nájera, Karina Yew-Hoong Gin, Jennifer L. Graham, Dominik Strapagiel, Rebecca M. Gorney, Jerome Wai Kit Kok, Shu Harn Te, Magdalena Kluska, Milena Skóra, Michał Seweryn, Francisco López-Hun

**Affiliations:** 1 Faculty of Biology and Environmental Protection, UNESCO Chair on Ecohydrology and Applied Ecology, University of Lodz, Lodz, Poland; 2 European Regional Centre for Ecohydrology of the Polish Academy of Sciences, Lodz, Poland; 3 Department of Civil and Environmental Engineering, National University of Singapore, Singapore, Singapore; 4 U.S. Geological Survey, Troy, NY, United States; 5 Faculty of Biology and Environmental Protection, Centre for Digital Biology and Biomedical Science - Biobank Lodz, University of Lodz, Lodz, Poland; 6 Doctoral School BioMedChem, University of Lodz and Institutes of the Polish Academy of Sciences, Lodz, Poland

**Keywords:** cyanosphere, coccoidal cyanobacteria, filamentous cyanobacteria, diazotrophic cyanobacteria, non-diazotrophic cyanobacteria, pathogenic bacteria, nutrient-cycling genes

## Abstract

Cyanobacterial toxicity, cyanotoxins, and their impact on aquatic ecosystems and human health are well documented. In comparison, less is known about bloom-associated bacterial communities. Co-occurring bacteria can influence bloom development, physiology and collapse, and may also provide a niche for pathogenic bacteria. Existing research focuses on the cyanosphere of *Microcystis*-dominated blooms, despite the increasing prevalence of filamentous genera (*Aphanizomenon* and *Planktothrix*). This pilot study aimed to broaden our understanding of the bacterial consortia attached to morphologically distinct cyanobacteria (coccoid and filamentous) dominating phytoplankton communities and to explore their potential roles in amplifying the impacts of cyanobacterial blooms. We investigated four shallow freshwater bodies across three continents and two climate zones: an urban pond in the USA, a dammed reservoir and a natural lake in Poland, and an urban water body in Singapore. Amplicon sequencing (16S rRNA gene) was used to characterize bacterial communities, while shotgun metagenomics identified nitrogen- and phosphorus-cycling genes to infer potential eco-physiological functions. Cyanobacteria dominated bacterioplankton assemblages at all sites (>35.6%), with bloom composition influencing toxigenic profiles. A mixed bloom of *Microcystis*, *Snowella*, and *Aphanizomenon* had the broadest range of cyanotoxin synthetase genes (*mcy*E, *cyr*J, *ana*F and *sxt*A). *Microcystis* blooms correlated with increased *Roseomonas*, while *Planktothrix* co-occurred with *Flavobacterium* – both bacteria likely contribute to nutrient-cycling within blooms and represent potential opportunistic pathogens for aquatic organisms and humans. The *Microcystis* cyanosphere exhibited the highest number of significant positive correlations with bacteria (19 relations), compared to *Planktothrix* and *Aphanizomenon* (11 and 2 relations, respectively). Non-diazotrophic blooms of *Microcystis* and *Planktothrix* showed greater abundances of nitrogen – (*ure*B, *gln*A, *nar*B, and *nar*HZ) and phosphorus-cycling genes (*pho*BHPR and *ppk*1), indicating a strong dependence on associated bacteria for nutrient acquisition compared to diazotrophic *Aphanizomenon*. These findings suggest that *Aphanizomenon*-dominated blooms may be sustained by simpler microbiomes. Our results provide preliminary evidence of cyanosphere heterogeneity potentially shaped by the dominance or coexistence of three morphologically and eco-physiologically distinct genera of cyanobacteria. A comprehensive knowledge of the taxonomy and functional roles of bloom-associated microbiomes is therefore essential to understand bloom activity, evaluate the environmental threat, and develop effective strategies for prevention and mitigation.

## 1 Introduction

Harmful cyanobacterial blooms (HCBs) are common threats resulting from the eutrophication of freshwater ecosystems, a situation exacerbated by increased anthropogenic development (anthropopressure) and global climate change (Paerl, 2014; Paerl and Barnard, 2020; Burford et al., 2020). Cyanotoxins are pollutants produced by cyanobacteria that represent a potential hazard to human and aquatic ecosystem health and include hepatotoxic microcystins (MCs) and cylindrospermopsins (CYN), and neurotoxic anatoxins (ATX) and saxitoxins (SXT) (Svirčev et al., 2019). Blooms dominated by coccoidal colonies of *Microcystis* spp. are among the most well documented due to their ubiquitous presence worldwide and their ability to produce MCs – the most common and well-studied cyanotoxin (Harke et al., 2016). Recent studies have reported that HCBs in freshwater ecosystems are shifting from *Microcystis*-dominated populations to other types of filamentous cyanobacteria, such as *Aphanizomenon* spp. (Parulekar et al., 2017; Zhang et al., 2020) and *Planktothrix* spp., both of which can produce CYN, ATX, and SXT (Wang et al., 2021). The toxicity of CYN (LD_50_ = 6.9 μg g^−1^), ATX (LD_50_ = 20–200 μg g^−1^) and SXT (LD_50_ = 10 μg g^−1^) has been reported to be as high or higher than that of MCs (LD_50_ = 50–600 μg g^−1^) (Christensen and Khan, 2020). However, there are few studies focusing on microbial and ecological factors associated with the domination of HCBs by filamentous forms of cyanobacteria. Therefore, additional studies could help identify other factors that elevate the environmental and health threats related to HCBs beyond our knowledge of their potential toxigenicity.

Interactions of bacteria with cyanobacteria (interactome) are important elements that alter the ambient chemical composition, physiology, and development of HCBs in freshwater ecosystems (Cook et al., 2020; Mankiewicz-Boczek and Font-Nájera, 2022). The bacteria thrive embedded in the surrounding extracellular polysaccharide space of cyanobacterial cells (EPS mucilage), known as the cyanosphere, where synergistic and antagonistic interactions occur that can alter the fate of the blooms (Le et al., 2022a). These interactions can shape the ecosystem diversity of bacterioplankton, including the dominance of cyanobacterial taxa in phytoplankton communities (Cook et al., 2020). Recent studies have investigated the diversity of bacteria attached to cyanobacteria. However, most have been focused on HCBs dominated by *Microcystis* spp. (Le et al., 2024). There is a lack of information regarding blooms that are dominated by filamentous cyanobacteria, such as *Aphanizomenon* spp. and *Planktothrix* spp. *Microcystis* blooms have been found to contain a high diversity of associated bacterial communities because they produce thick mucilage with rich EPS substances (Le et al., 2022a). Only a few studies have described the associated bacterial communities of *Aphanizomenon* spp. (Parulekar et al., 2017; Underwood et al., 2024) or *Planktothrix* spp. (Wang et al., 2021) and selected potential ecological functions. These studies reported that *Flavobacterium* and *Rheinheimera* were highly abundant in *Aphanizomenon*-dominated blooms, while *Flavobacterium* was also prominent in a *Planktothrix*-dominated bloom. However, these bacterial communities have not been directly compared with those associated with *Microcystis*-dominated blooms. There is a need to address this knowledge gap, since filamentous cyanobacteria (e.g., *Aphanizomenon*, *Pseudanabaena*, *Dolichospermum* and *Raphidiopsis*) are expected to occur more frequently in the context of global climate change and increasing anthropopressure, potentially outcompeting species like *Microcystis* (Gao et al., 2025).

HCBs can harbor pathogenic microorganisms, which is attributed to the ability of cyanobacteria to develop dense colonies in surface waters. Berg et al. (2009) isolated many pathogenic bacterial strains, e.g., *Aeromonas*, *Vibrio*, *Acinetobacter* and *Pseudomonas* spp., from colonial forms of cyanobacteria obtained from different Nordic lakes and the Baltic Sea. Unfortunately, the dominant cyanobacterial genera in these phytoplankton communities were not reported. To our knowledge, the presence of pathogenic bacteria has not been well characterized for HCBs dominated by coccoidal forms of *Microcystis* or filamentous forms of *Aphanizomenon* and *Planktothrix*. All three cyanobacteria are known to form dense colonial aggregations in surface waters, which indicates their potential ability to harbor these organisms.

Cyanobacteria are known to exhibit diverse nutrient affinities and nutrient cycling strategies, including phosphorus (P) and nitrogen (N). Non-diazotrophic cyanobacteria, such as *Microcystis* spp., have a higher affinity for ammonium and adapt well to N-rich environments (Bell et al., 2018). *Microcystis* spp. have higher P-affinity, that allows them to better thrive under P-limiting conditions than many other cyanobacteria (Harke et al., 2012). Non-diazotrophic cyanobacteria, such as *Planktothrix* spp., are well adapted to N- and P-rich environments, and can persist year-round in temperate freshwater bodies (Kokociński et al., 2011; Mankiewicz-Boczek et al., 2011; Wejnerowski et al., 2024). Conversely, diazotrophic cyanobacteria, such as *Aphanizomenon* spp. thrive in N-limiting conditions because of their ability to fix gaseous N (De Nobel et al., 1995; De Nobel et al., 1997). In summary, *Microcystis*, *Planktothrix* and *Aphanizomenon* may use different strategies for nutrient utilization, but these strategies are not well understood in the context of their dominance or co-occurrence in blooms.

Attached bacteria can play important roles in the transformation of unavailable nutrients into forms accessible to cyanobacteria during the development of HCBs (Yang et al., 2021; Wan et al., 2022; Yan et al., 2023). It has been hypothesized that bacteria may influence the biological strategies of cyanobacteria to cope with nutrient limitations in the environment. Nevertheless, their roles have been scarcely described in the literature, and most research has focused on intraspecific strategies of cyanobacteria. *Microcystis* blooms have been associated with high abundances of bacterial nitrogen decomposition genes (glutamate synthesis) and assimilatory and dissimilatory nitrate reduction to ammonium genes (ANRA and DNRA), regarded as aiding *Microcystis* spp. to utilize ammonium in N-rich environments. *Microcystis* blooms were also linked to high abundances of bacterial P-solubilizing genes, such as alkaline phosphatases, which are important for P transformation and availability in P-limiting environments (Yang et al., 2021; Wan et al., 2022; Yan et al., 2023; Cai et al., 2024). Wang et al. (2021) identified that *Planktothrix*, in a bloom co-dominated with *Microcystis*, was associated with the expression of microbial genes involved in N-synthesis and decomposition and alkaline phosphatases, which was attributed to a N-rich and P-limiting environment. To our knowledge, there are no studies describing the role of associated bacteria and availability of nutrients in blooms dominated by *Aphanizomenon* spp. The limited knowledge on the role of bacteria in the transformation and availability of nutrients highlights the need for further investigation, especially because nutrients play a key role in the development of HCBs (Paerl and Barnard, 2020).

The present study aimed to increase our knowledge of bacterial community diversity and their potential roles influencing the threat of HCBs dominated or mixed by coccoidal *Microcystis* or filamentous *Aphanizomenon* and *Planktothrix*. Our freshwater study sites are in three different countries: The Lake in Central Park (USA; U.S. Geological Survey station 404630073580801; U.S. Geological Survey, 2025), Raczyńskie Lake and Sulejów Reservoir (Poland), and an urban water body in Singapore. This study is preliminary, and the findings presented here are intended to provide baseline information for future comparative analyses. The investigation will be expanded through increased sampling efforts and inclusion of additional freshwater bodies, in accordance with the objectives of the ongoing CyMiBiom project (2024-2025). We focused on describing four elements that may increase the threat of HCBs to environmental health: (i) cyanobacterial toxigenicity, (ii) the diversity of associated bacterial taxa, (iii) presence of pathogenic bacteria, and (iv) abundance of bacterial nutrient-cycling genes. These elements will enhance our understanding of the eco-physiological roles of bacterial communities, allowing the development of hypotheses about the diverse relationship between three different cyanobacterial genera and their attached consortia. To accomplish our objectives, amplicon sequencing with the 16S rRNA gene was used to characterize bacterioplankton communities and shotgun metagenomic constructs were used to mine for the presence of N- and P-cycling and toxigenic genes. This knowledge is essential to understand the pervasive threat of variable HCBs in freshwater ecosystems worldwide.

## 2 Materials and methods

### 2.1 Study sites and sample processing

Four freshwater bodies, known to harbor different communities of bloom-forming cyanobacteria, were selected for our study (Supplementary Figure S1). The Lake in Central Park (site TL) in the City of New York, New York, is within the most visited park in the USA and has a high density of walking trails (Fisher, 2011). The Lake is an artificial impoundment used for recreational boating by thousands of people annually. Near shore monitoring in the last decade has shown that annual summer HCBs are highly toxic and dominated by *Microcystis viridis* (Flanzenbaum et al., 2022). Raczyńskie Lake (site RA) in Poland is a natural freshwater body formed by the Kamionka River in western Poland that has gradually changed from an agricultural to a recreational area, with substantial tourism pressure. HCBs in Raczyńskie Lake have been reported to contain *Aphanizomenon flos-aquae*, *Planktothrix agardhii*, *Dolichospermum affine* and *Microcystis aeruginosa* (Kowalczewska-Madura et al., 2022). The Sulejów Reservoir (site SU) in Poland is in the Pilica River catchment in central Poland originally designated for power generation, flood control, recreation and as an alternative water supply for the City of Łódź (Izydorczyk et al., 2008). Sulejów Reservoir is known to experience intense summer HCBs of *Aphanizomenon flos-aquae* and *Microcystis* spp. (Jaskulska et al., 2021; Mankiewicz-Boczek and Font-Nájera, 2022). In Singapore, the selected freshwater body (site SP) is an urban reservoir (hereafter referred to as “Singapore urban reservoir”) constructed by damming a river mouth located near the city of Singapore. Singapore urban reservoir is used as a source of drinking water supply and for recreational activities such as fishing and picnicking. The phytoplankton communities in this reservoir have changed, shifting from *Microcystis*-dominated blooms to mixed assemblages of *Microcystis* and filamentous cyanobacteria, such as *Anabaena* (Te and Gin, 2011).

Water samples were collected in shallow (<2 m), nearshore areas at each site, 0–50 cm below the surface. Three surface grab samples (up to 1 L each) were taken from each water body and homogenized in a large vessel that was pre-rinsed several times with local freshwater. Homogenized water was stored in plastic containers (up to 3 L), that were previously washed with ethanol and deionized water and preserved in cold (4 °C) and dark conditions until transport to local laboratories. Sampling was conducted during hot and sunny conditions in 2023, which favored the visible green coloration of water and the formation of cyanobacterial surface scums (Supplementary Figure S2). For the water bodies located in temperate zones, sampling took place at the end of the summer season on September 5th for RA, and on September 20th for SU and TL. In contrast, sampling at SP, which is located in the tropical zone was carried out on November 16th, coinciding with the end of an inter-monsoon period (Table 1). Physico-chemical parameters were measured on site using a multiparameter probe (YSI EXO2, YSI, Inc., Yellow Springs, OH, USA), including temperature, pH, dissolved oxygen concentration and specific conductance. Samples were processed for different analyses immediately upon return to the local laboratories. For dissolved nutrient composition, water was filtered through GF/C Whatman filters (0.45 μm) and the filtrate was frozen (−20 °C) until further analysis at local laboratories. For DNA analysis, 30–100 mL of surface water were filtered through 1.2 μm mixed cellulose ester (MCE) filters, in triplicate, to collect the particle-attached microbial fraction (Mankiewicz-Boczek and Font-Nájera, 2022). Laboratory controls, in duplicate, were prepared with the filtration of 100 mL of deionized water through 0.22 μm MCE filters at each local laboratory. These controls were used to describe background contamination, such as bacterial DNA in deionized water or laboratory materials and surfaces. Filters for DNA analysis were dried for 1 hour in aseptic conditions and shipped to Poland in ambient temperature for further analyses.

**Table 1:** Surface water environmental parameters of studied sites.

Country	Site	Sampling date	Temp. (°C)	pH	Sp. Cond. (μS cm^−1^)	Oxygen (mg L^−1^)	N-NH_4_^+^	N-NO_3_^−^	N-NO_2_^−^	P-PO_4_^3−^
							(μg L^−1^)			
USA	The Lake in Central Park (TL)*	20.09.2023	20.5	8.3	191.1	9.91	bd	bd	bd	105.60
Poland	Raczyńskie Lake (RA)	05.09.2023	23.6	8.3	538.0	8.47	9.59	34.16	0.030	15.62
Poland	Sulejów Reservoir (SU)	20.09.2023	23.6	7.3	267.6	9.18	28.69	36.87	0.053	1.53
Singapore	Urban Reservoir (SP)	16.11.2023	30.2	8.0	212.0	8.67	bd	bd	bd	34.00

Temp, temperature; Sp. Cond, specific conductance; bd, below limit of detection. *U.S. Geological Survey station number 404630073580801 (U.S. Geological Survey, 2025).

### 2.2 Nutrient analyses

Water samples were immediately filtered and processed at local laboratories. For TL, orthophosphate (P-PO_4_^3−^) concentration was measured at the U.S. Geological Survey National Water Quality Laboratory (Denver, CO, USA) by discrete analyzer phosphomolybdate formation and colorimetry (detection limit of 4 μg L^−1^), according to Fishman (1993). Nitrogen species were analysed as N-NH_4_^+^ with the salicylate-hypochlorite colorimetry method (detection limit of 20 μg L^−1^), and NO_3_^−^ + NO_2_^−^ with the diazotization colorimetry method (detection limit of 40 μg L^−1^), according to Fishman (1993). In Poland and Singapore, ion chromatography was used to estimate the concentration of above-mentioned nutrients according to Gągała et al. (2014) (limit of detection for all nutrients was 1 μg L^−1^).

### 2.3 DNA isolation

Filters containing the particle-attached microbial fraction were placed into separate bead tubes and DNA was extracted with the specifications in the Dneasy® PowerWater® Kit (Qiagen, Hilden, Germany). DNA quality and quantity were measured with a NanoDrop microvolume spectrophotometer (Thermo Fisher Scientific, Waltham, MA, USA) and stored at −20 °C for further analyses.

### 2.4 Sequencing analyses

#### 2.4.1 16S rRNA amplicon sequencing

DNA libraries (16S rRNA) were prepared with a 25 μL Polymerase Chain Reaction (PCR) mixture containing 12.5 μL of 2 x Phanta Master Mix (Vazyme, China), 2.5 μL of DNA template (5 ng μL^−1^), 0.5 μL of primers (10 μM μL^−1^) specific to V3-V4 regions of the 16S rRNA gene (Klindworth et al., 2013), designed with Illumina overhang adapters following the 16S Metagenomic Sequencing Library Preparation Guide (Illumina, 2023). Amplicon PCR conditions included 95 °C for 3 min, 25 cycles of 95 °C for 30 s, 55 °C for 30 s and 72 °C for 30 s, and a final extension of 72 °C for 5 min. PCR products were cleaned with Sera-Mag beads (Cytiva, UK) and analysed by electrophoresis on 1.5% agarose gel. PCR products were indexed with Nextera XT index kit (Illumina, Inc., San Diego, CA, USA) using the above-mentioned specifications for PCR with an 8-cycle amplification program. Products were quantified by quantitative PCR (qPCR) using NEBNext Library Quant kit for Illumina (New England Biolabs, Inc., Ipswich, MA, USA). Libraries were sequenced (2 × 250 bp paired-end reads) using NovaSeq 6000 SP Reagent kit (Illumina, USA) on the Novaseq 6000 platform (Illumina, USA) with an expected amount of 100,000 pair-end reads per sample. Amplicon sequencing was prepared in triplicates for each site.

#### 2.4.2 Shotgun metagenomics

DNA libraries were prepared with the NexteraXT DNA library preparation kit (Illumina, USA) according to the manufacturer’s instructions (Illumina, 2021). The quality of libraries was assessed with Agilent Technologies (Santa Clara, CA, USA) Bioanalyzer 2,100 system with Agilent High Sensitivity DNA kit. Libraries were normalized with qPCR, similarly, as performed with amplicon sequencing. Metagenomics were performed by shotgun sequencing concurrently in the same Illumina platform during amplicon sequencing, also for 2 × 250 bp paired-end reads and maximum assumption of 50 million paired reads per sample. Shotgun metagenomics was prepared in duplicate for each site, reflecting the pilot nature of this study and the higher resource demands of shotgun sequencing relative to 16S rRNA amplicon sequencing.

#### 2.4.3 Bioinformatic analyses

For 16S rRNA analysis, reads were edited with the package DADA2 1.32 (Callahan et al., 2016) in R environment 4.3.2 (R core Team, 2023), including filtering, trimming, error analysis, merging, chimera removal and allocation of taxa. The SILVA 138 database was utilized for amplicon sequence variants (ASV) taxonomical assignation by similarity (≥97% homology) to bacteria.^1^ Sequences assigned to mitochondria and chloroplasts were removed. The phyloseq package 1.42 was used for data visualization (McMurdie and Holmes, 2013). Original datasets were uploaded to the NCBI Sequence Read Archive^2^ as FASTQ files under the project PRJNA1241204, with three biosamples for TL (SAMN47547428 – SAMN47547430), RA (SAMN47547431 – SAMN47547433), SU (SAMN47547434 – SAMN47547436) and SP (SAMN47547437 – SAMN47547439).

For shotgun metagenomic analysis, sequences were trimmed to reduce sample contamination – a palindromic algorithm was used for sequence alignment with appropriate adaptors (TrimmomaticPE v0.39 + dfsg-2; Bolger et al., 2014). Trimmed sequences were merged using Vsearch command, and shotgun metagenomes were mined for the presence of cyanotoxin-producing genes and nutrient-transforming genes (N- and P-cycling genes) using BLAST via the DIAMOND tool in the conda environment for Python 2.7.18 (Python Software Foundation, 2020). For cyanotoxin synthesis, genes involved in microcystins (*mcy*E), cylindrospermopsins (*cyr*J), anatoxins (*ana*C), and saxitoxins (*sxt*A) synthesis were included. Cyanotoxin genes were utilized to describe the potential toxigenicity of cyanobacterial blooms. For nitrogen (N) – cycling, several genes involved in N-decomposition and ammonium assimilation (*ure*B, *gln*A, *gcv*T, *glt*B, *glt*D, and *amt*), nitrification (*amo*A, *nxr*A, and *nxr*B), complete nitrification COMAMMOX (_c_*amo*A), anaerobic nitrification ANAMMOX (*hzs*A and *hzo*), assimilatory or dissimilatory nitrate reduction to ammonium – ANRA and DNRA (*nap*A, *nar*G, *nar*Z, *nar*H, *nar*B, *nrf*A, and *nrf*H), denitrification (*nir*S, *nir*K, *nor*B, *nor*R, and *nos*Z) and nitrogen fixation (*nif*K and *nif*B) were included. For phosphorus (P) – cycling, genes involved in phosphorus solubilization (*pho*B, *pho*R, *pho*D, *pho*H, *pho*L, and *pho*P), phosphorus accumulation in the form of polyphosphate (*ppk*1), and polyphosphate solubilization and release of phosphorus (*ppx*) were included. The genes were used to describe potential eco-physiological roles of bacterioplankton communities in nutrient-transformation processes. Gene accession numbers are summarized in Supplementary Table S1. Gene counts (hit numbers) were filtered according to recommended parameters (sequence length ≥25 aa, match score ≥50 and e-value ≤1 × 10^−10^) and normalized to the 16S rRNA gene count pooled from shotgun metagenomic datasets. Original datasets as FASTQ files were uploaded to the NCBI Sequence Read Archive^2^ under the project PRJNA1241753, with two biosamples for TL (SAMN47568682 – SAMN47568683), RA (SAMN47568684 – SAMN47568685), SU (SAMN47568686 – SAMN47568687) and SP (SAMN47568688 – SAMN47568689).

### 2.5 Data analyses

Rarefaction curve analysis was employed to estimate the sequencing depth of amplicons. The indices Shannon-Weiner (H′), abundance-based coverage estimator (ACE) richness, evenness (*J*) and Simpson’s dominance (*D*) were used to describe the diversity of bacterioplankton communities (refer to Supplementary Table S2). Principal component analysis (PCA) was used to describe differences between study sites based on the bacterioplankton communities and the composition of nutrient-cycling genes. Due to the large amount of ASVs, PCA was constructed with the taxa that contributed with the highest abundances (>1% of relative abundance per sample). Significant differences in diversity indices and spatial ordination of the samples were described with Kruskal–Wallis and Dunn’s *post hoc* tests (*p* < 0.05). Spearman’s correlation (*r*) was used to describe the relationships within the microbiological community (cyanobacteria and other bacteria). Bonferroni corrections were applied to *p*-values to reduce the finding of false positive and negatives. Graphs were prepared with the statistical software PAST 4.12b (Hammer et al., 2001).

## 3 Results

### 3.1 Environmental characterization of surface waters

Physico-chemical parameters and nutrient composition varied across water bodies (Table 1). The temperature showed clear differences according to latitude, with temperatures ranging from 20.5 to 23.6 °C for temperate water body sites (TL, RA and SU) and to the highest of 30.2 °C for the tropical site SP (Table 1). The generally observed basic pH (7.3–8.3) and high concentrations of dissolved oxygen (8.47–9.91 mg L^−1^) in all water bodies are typical during daylight hours in surface waters containing HCBs (Table 1). Specific conductance was more variable, with the highest values observed in the water bodies located in Poland (RA = 538.0 μS cm^−1^ and SU = 267.6 μS cm^−1^) (Table 1). In the case of nutrients, the TL, SP and RA were characterized with the highest P-PO_4_^3−^ concentrations (105.6, 34.00 and 15.62 μg L^−1^, respectively) (Table 1). Notably, TL, SP and RA were an order of magnitude higher than observed in SU (1.53 μg L^−1^ of P-PO_4_^3−^), and concentrations in TL were about 3 to 7 times higher than in SP and RA, respectively (Table 1). All nitrogen species were below the limit of detection in TL and SP (Table 1). In contrast, concentrations were much higher in the Polish sites (RA and SU). The N-NO_x_ concentrations were similar in RA and SU (34.16 and 36.87 μg L^−1^ of N-NO_3_^−^_,_ and 0.030 and 0.053 μg L^−1^ of N-NO_2_^−^, respectively; Table 1), but N-NH_4_^+^ concentration was about 3-times higher in SU than RA (28.69 and 9.50 μg L^−1^, respectively; Table 1).

### 3.2 Description of toxic cyanobacterial communities

**Figure 1:**
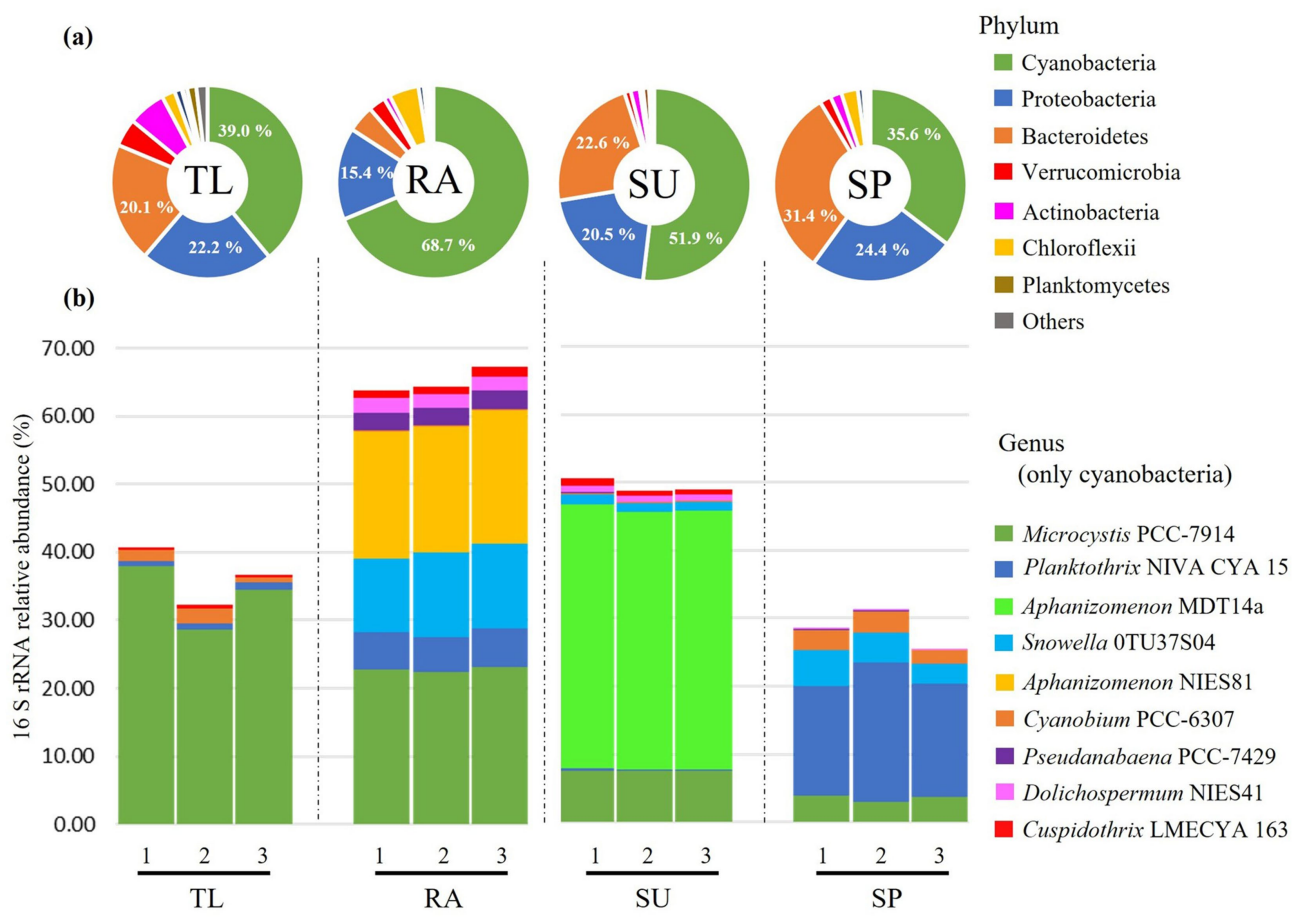
Relative abundances (%) of bacterial communities using 16S rRNA amplicon sequencing. Abundances were described to the level of phylum **(a)**, and in the case of genus, cyanobacteria were displayed separately from other types of bacteria **(b)**. Unclassified sequences and other taxa with lower relative abundance (<1%) were excluded in **(b)**. Relative abundances were estimated for each study site (TL: The Lake in Central Park – New York, USA; RA: Raczyńskie Lake, Poland; SU: Sulejów Reservoir, Poland; SP: Singapore urban reservoir) using sample replicates (*n* = 3).

A database containing 3,512,878 raw reads was obtained from 12 samples using 16S rRNA amplicon sequencing. A total of 2,780,855 good quality sequences were retrieved after standard quality control, of which 2,661,437 sequences were classified to bacteria (Supplementary Table S3). Classified sequences were composed of 6,798 ASVs belonging to 50 phyla, 107 classes, 227 orders, 309 families and 540 genera. Rarefaction curve analysis showed that all samples reached a plateau, indicating that the sequencing depth was adequate to describe overall bacterial diversity (Supplementary Figure S3). Laboratory controls were estimated to contain <75 copies of 16S rRNA gene per ng DNA using qPCR, which represents roughly 3.38 × 10^−4^% of the average number of good quality reads with assigned taxonomy used in the present study (Supplementary Table S3). DNA libraries were not obtained from laboratory controls due to low DNA material (<3.1 ng uL^−1^); therefore, we expected minimal background laboratory contamination. Classified reads were shown as relative abundances of the 16S rRNA gene representing different bacterioplankton communities (Figure 1). Generally, bacterial communities were dominated by Cyanobacteria (35.6–68.7%), followed by Proteobacteria (15.4–24.4%) and Bacteroidetes (4.5–31.4%) (refer to Figure 1a; Supplementary Table S4). Despite cyanobacterial dominance in all samples, cyanobacterial community composition differed among study sites (Figure 1b; Supplementary Table S5). TL had the highest relative abundance of *Microcystis* PCC-7914 (~ 33.4%), while the other sites were mixed between filamentous and coccoidal forms of cyanobacteria (Figure 1b; Supplementary Table S5). RA had high relative abundance of *Microcystis* PCC-7914 (~22.8%), followed by *Aphanizomenon* NIES81 (~18.9%) and *Snowella* 0TU37S04 (~11.9%) (refer to Figure 1b; Supplementary Table S5). The cyanobacterial composition in SU was dominated by *Aphanizomenon* MDT14a (~38.2%) and had lower abundance of *Microcystis* PCC-7914 (~7.5%) (refer to Figure 1b; Supplementary Table S5). Finally, SP had the highest relative abundance of *Planktothrix* NIVA CYA 15 (~17.6%), mixed with lower amounts of *Snowella* 0TU37S04 (~4.2%) and *Microcystis* PCC-7914 (~3.6%) (refer to Figure 1b; Supplementary Table S5). *Microcystis* PCC-7914 was the only cyanobacterial taxon commonly shared among all study sites.

**Figure 2:**
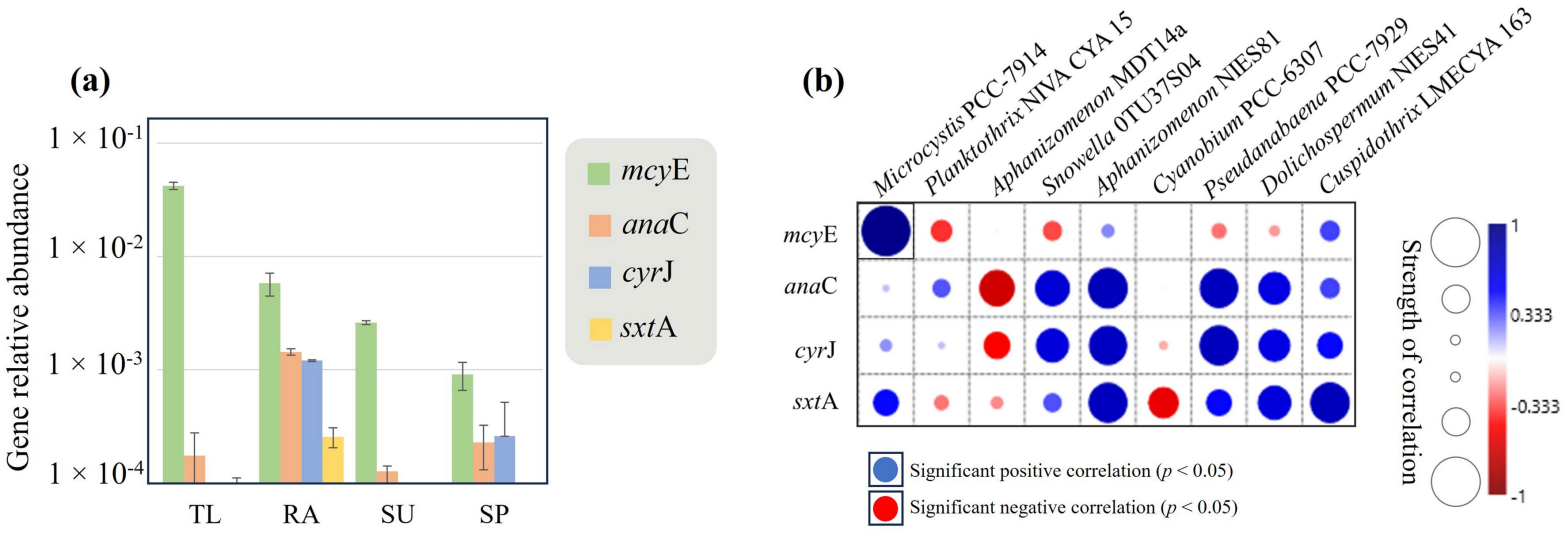
The overall relative abundance of cyanotoxin synthetase genes normalized to the total number of 16S rRNA **(a)**, and their relation (non-parametric Spearman’s correlation) to the abundance of cyanobacterial taxa in **(b)**. Bars and error lines were estimated for each study site (TL: The lake in Central Park – New York, USA; RA: Raczyńskie Lake, Poland; SU: Sulejów Reservoir, Poland; SP: Singapore urban reservoir) using sample replicates (*n* = 2).

On average, a total of 2.65 × 10^7^ reads per sample were obtained from shotgun metagenomes, with good quality reads reaching 1.68 × 10^7^. Total merged reads were 32.3–50.5% of good quality reads, which were used for formal analyses. Editing features and error statistics for shotguns are presented in Supplementary Table S6. A total of 2,000 hit numbers, representing selected cyanotoxin genes, were obtained from shotguns and normalized to the 16S rRNA (refer to Supplementary Table S7). Potential toxigenicity of HCBs in selected study sites was also described (Figure 2). Overall, *mcy*E gene counts were notably higher than other cyanotoxin genes at all sites. TL had the highest *mcy*E gene relative abundance compared to the other study sites, followed by RA, SU and, finally, SP (Figure 2a). Correlation analysis revealed that *Microcystis* PCC-7914 was the only cyanobacterial taxon that positively correlated with the normalized abundance of *mcy*E (*r* = 0.96, *p* = 0.047) (refer to Figure 2b; Supplementary Table S8). The genes *ana*C, *cyr*J and *sxt*A were all highest at RA compared to the other sites. RA was the only site where all four cyanotoxin genes were detected, and that *cyr*J was not detected in TL and SU, and *sxt*A was not detected in SU and SP (Figure 2a). Strong positive correlations were observed between the relative abundance of *Aphanizomenon* NIES81 and normalized gene counts of *ana*C (*r* = 0.76), *cyr*J (*r* = 0.73), and *sxt*A (*r* = 0.75), although these were not significant after Bonferroni correction (Figure 2b; Supplementary Table S8). *Pseudanabaena* PCC7929 and *Cuspidothrix* LMECYA 163 correlated positively to genes *ana*C, *cyr*J, and *sxt*A, but were not considered for further analysis because they were detected in contrasting lower abundances (up to 2.6 and 1.2%, respectively) and were not significant after *p-*value correction.

### 3.3 Description of bacterial communities associated with cyanobacteria

**Figure 3:**
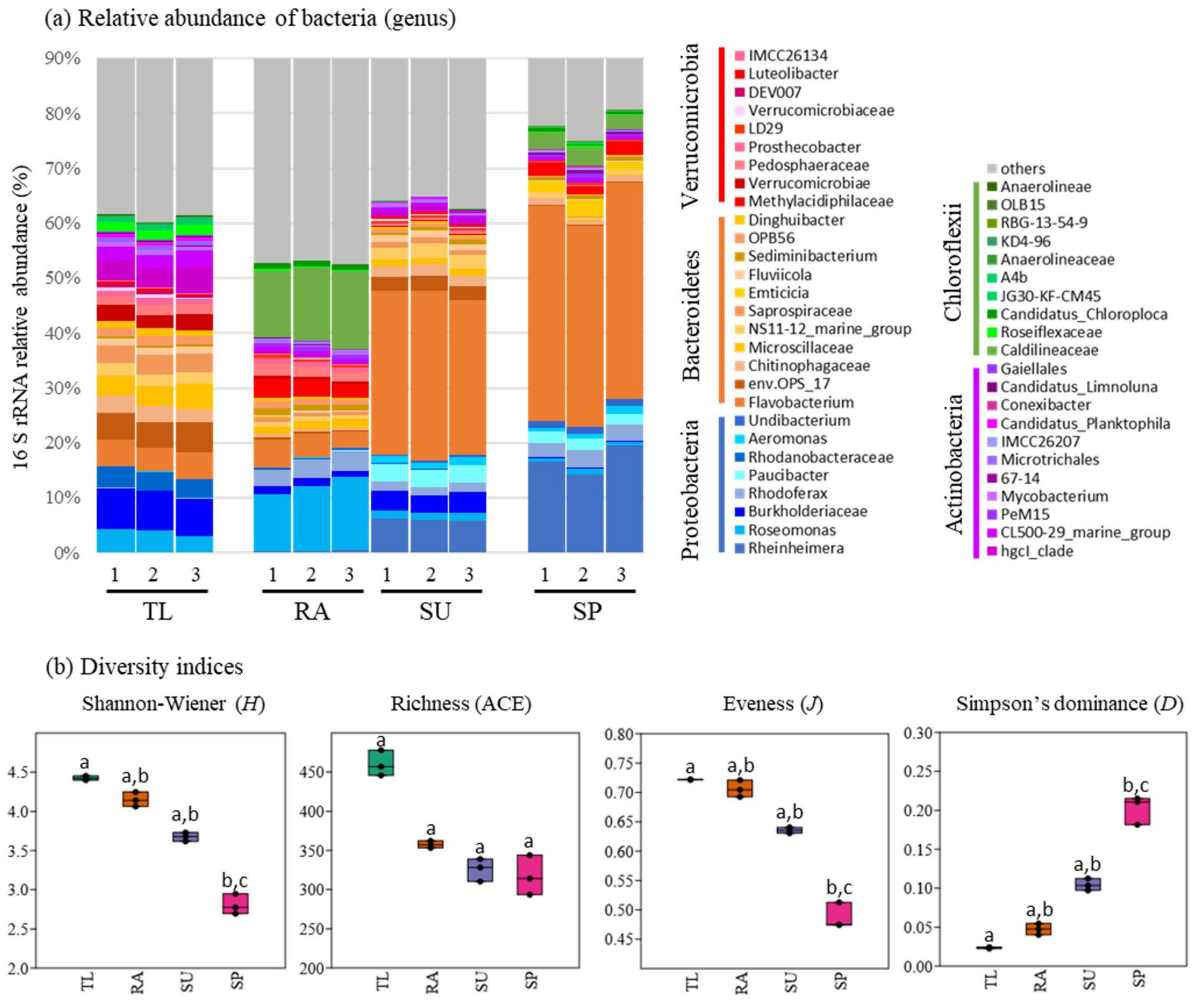
Abundance and diversity of bacterial communities associated with cyanobacteria through the use of 16S rRNA amplicon sequencing. Bacteria not belonging to cyanobacteria were displayed to the level of genus **(a)** in triplicate samples (*n* = 3), and diversity indices **(b)** using averaged samples (*n* = 3). Analysis was conducted to the lowest reliable taxonomic rank (genus level). Higher-level taxa indicate unclassified members at the corresponding taxonomic rank. Unclassified sequences and other taxa with lower relative abundance (<1%) were excluded in **(a)** for better visualization, while all classified data were utilized for diversity analysis in **(b)**. Significant differences among sites (TL: The Lake in Central Park – New York, USA; RA: Raczyńskie Lake, Poland; SU: Sulejów Reservoir, Poland; SP: Singapore urban reservoir) were estimated with non-parametric Kruskal–Wallis and Dunn’s *post hoc* test (*p* < 0.05). Sites with the same case letters (a–c) presented no significant differences.

The composition and diversity of bacterial communities – other than cyanobacteria – are shown with the use of the 16S rRNA amplicon sequencing (Figure 3; Supplementary Tables S9–S11). The composition of bacteria appeared more evenly distributed and diverse at TL compared to the other sites (Figure 3a). In fact, TL showed the highest values of Shannon-Wiener diversity (*H* = 4.4 ± 0.02), richness (ACE = 460.3 ± 13.3) and evenness (*J* = 0.72 ± 0.0002) (Figure 3b). Bacterial richness at TL was notably higher than all other sites, including RA (ACE = 357.5 ± 3.7), SU (ACE = 325.9 ± 11.8), and SP (ACE = 317.2 ± 20.7) (Figure 3b). The lowest diversity and evenness were observed at SP (*H* = 2.8 ± 0.1 and *J* = 0.49 ± 0.02, respectively; Figure 3b). In contrast, for Simpson’s dominance, SP showed the highest index value (*D* = 0.203 ± 0.015), which was notably different from SU (*D* = 0.104 ± 0.006), RA (*D* = 0.048 ± 0.006), and TL (*D* = 0.024 ± 0.001) (Figure 3b). The dominance indices at SP and SU were attributed to high relative abundance of two bacterial taxa: *Flavobacterium* and *Rheinheimera* (38.4 and 16.8%, respectively, at SP and 29.5 and 6.0%, respectively at SU; Figure 1a). Finally, a higher dominance value for RA compared to TL was attributed to high relative abundances of an undetermined taxon of Caldilineaceae (13.1%) and *Roseomonas* (11.7%; Figure 3a). There were no evident dominant bacterial taxa for TL (Figure 3a). Despite clear differences in diversity indices across sites, after applying the Bonferroni correction, statistically significant differences were observed only between TL and SP across Shannon-Wiener diversity, evenness and Simpson’s dominance (*p* = 0.01341 for all three indices; refer to Figure 3b; Supplementary Table S11).

### 3.4 Relations between cyanobacterial and other bacterial taxa

**Figure 4:**
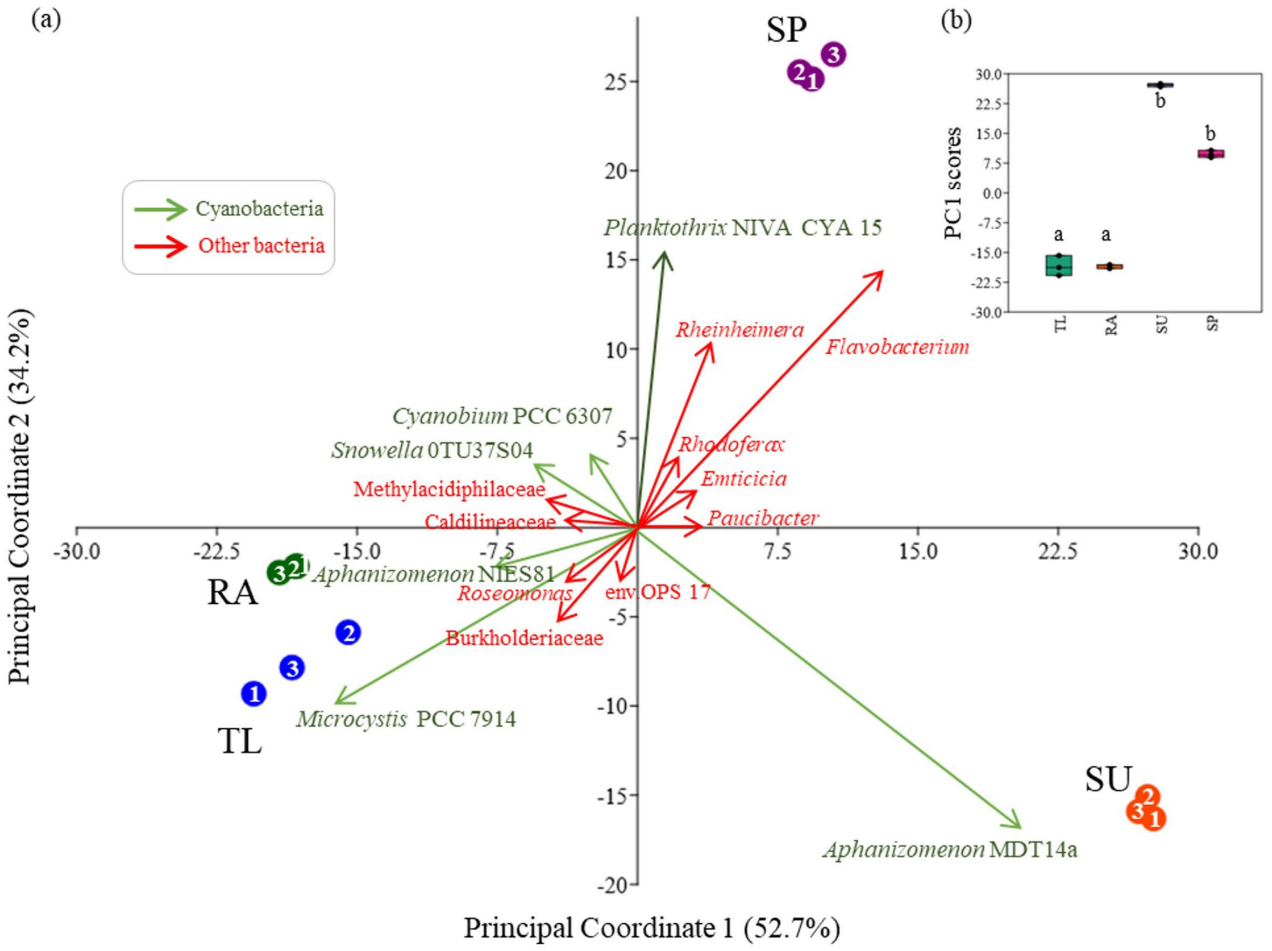
Principal component analysis (PCA) displaying the spatial distribution of study sites according to bacterioplankton composition **(a)**. Unclassified sequences and other taxa with lower relative abundance (<1%) were excluded for better visualization. Significant differences (*p* < 0.05) among sites were estimated with non-parametric Kruskal–Wallis and Dunn’s post hoc test for the PC1 scores **(b)**. Triplicate samples (1–3 of the same color) was utilized per study site (TL: The Lake in Central Park – New York, USA; RA: Raczyńskie Lake, Poland; SU: Sulejów Reservoir, Poland; SP: Singapore urban reservoir).

Differences in sites regarding the composition of bacterioplankton communities were described with principal component analysis (PCA; Figure 4). Scores and loadings for the construction of the PCA are presented in Supplementary Table S12, and the statistical analysis for the differentiation among sites in Supplementary Table S13. The horizontal axis (PC1–52.7% of variability) indicated significant differences in bacterioplankton communities for TL and RA when compared to SU and SP (Figure 4b). TL and RA where clearly distinguished by the cyanobacteria *Microcystis* PCC7914 and associated bacteria belonging to *Roseomona*s, an unclassified taxon of Burkholderiaceae and Sphingobacteriales_env.OPS17 (Figure 4a). RA was further differentiated from TL by the cyanobacterium *Aphanizomenon* NIES81 and other associated bacteria belonging to an unclassified taxon of Caldilineaceae (Figure 4a). SU was strongly differentiated solely by the cyanobacterium *Aphanizomenon* MDT14a, which was associated with *Paucibacter* (Figure 4a). The vertical axis (PC2–34.2% of variability) indicated that SP was the most different in bacterioplankton composition when compared to the other three sites (Figure 4a). This site was differentiated by *Planktothrix* NIVA CYA 15 and associated bacterial taxa belonging to *Rheinheimera* and *Flavobacterium*, and to a shorter extent, *Rhodoferax* and *Emticicia* (Figure 4a).

**Figure 5:**
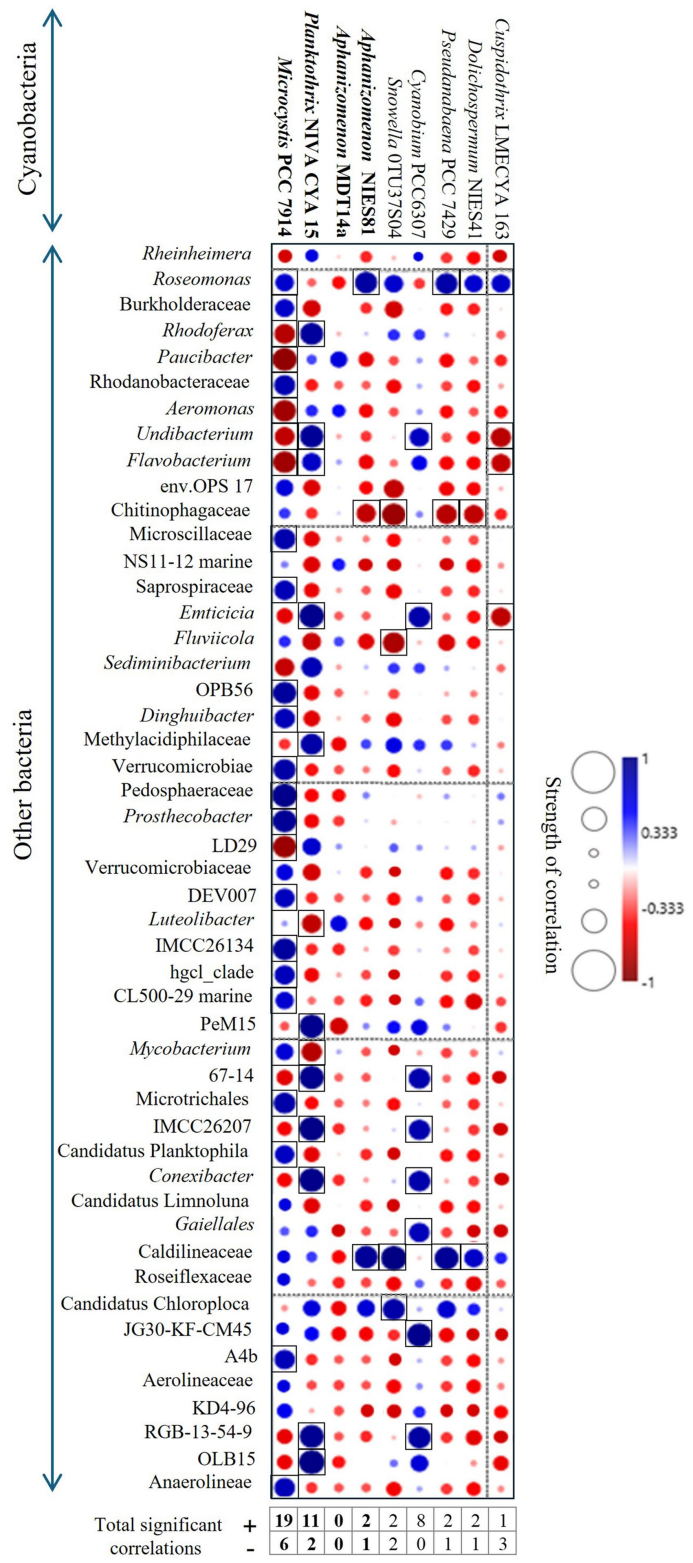
Relations between cyanobacterial and other bacterial taxa for the study sites. Non-parametric Spearman’s correlations between the relative abundance of each cyanobacteria strain and other bacteria were utilized. Analysis was conducted to the lowest reliable taxonomic rank (genus level). Higher-level taxa indicate unclassified members at the corresponding taxonomic rank.

Correlation analysis between cyanobacteria and associated bacterial taxa were described (Figure 5; Supplementary Table S14) and revealed that *Microcystis* PCC7914 and *Planktothrix* NIVA CYA 15 had the highest number of significant correlations with associated bacterial taxa (Figure 5; Supplementary Table S14). *Microcystis* strongly correlated with 25 non-cyanobacterial taxa, of which 19 were significant positive correlations (Figure 5). In contrast, *Planktothrix* NIVA CYA 15 strongly correlated with 13 non-cyanobacterial taxa, of which 11 were significant positive correlations (Figure 5). Fewer significant positive and negative correlations were observed for *Aphanizomenon* strain, NIES81 (2 and 1, respectively, Figure 5).

### 3.5 Nutrient-transforming genes in metagenomic constructs

**Figure 6:**
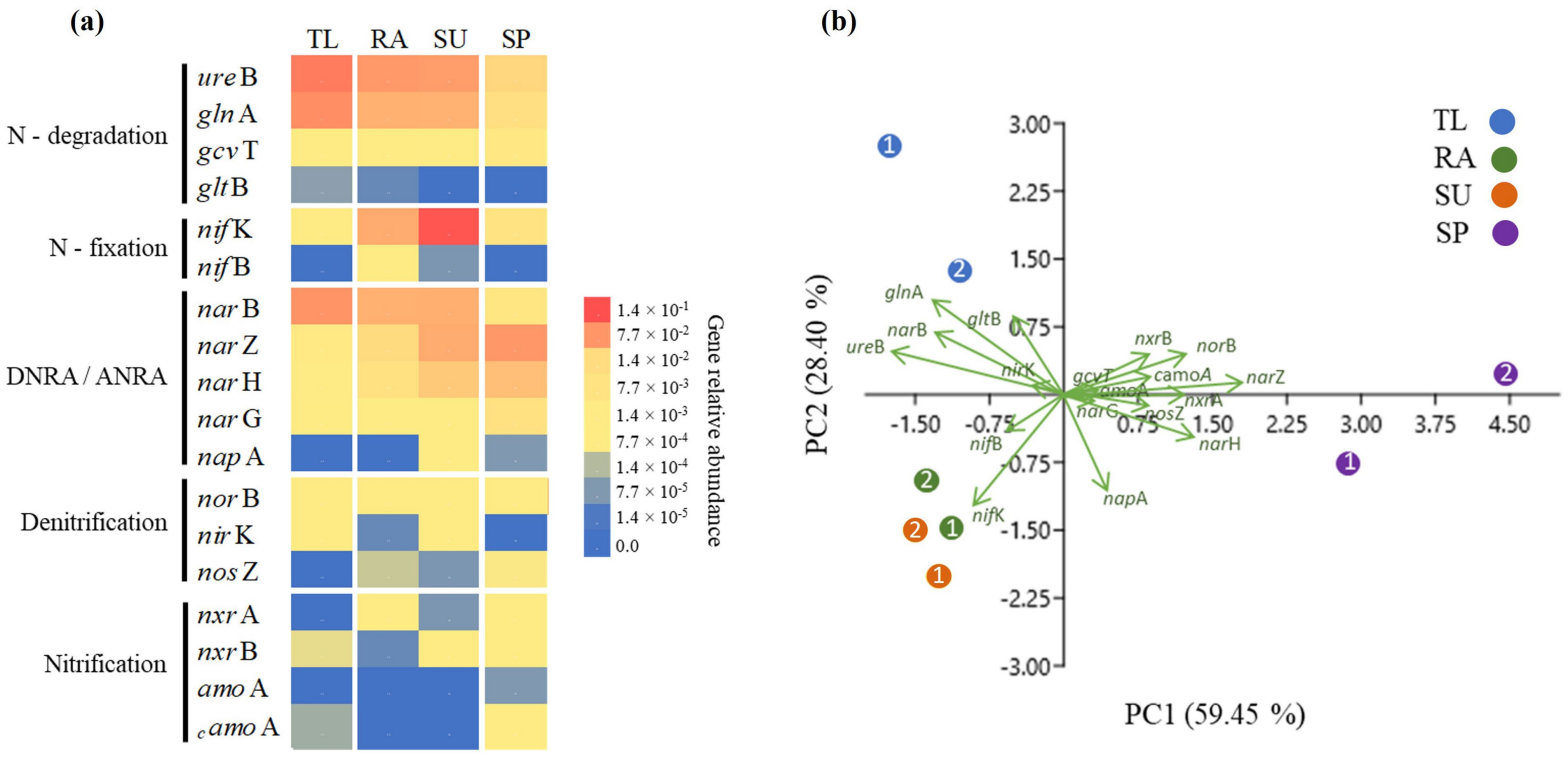
Relative abundances of nitrogen cycling genes in the study sites: **(a)** Heat map representing the differences in relative abundances of genes normalized with 16S rRNA of total bacteria; **(b)** principal components analysis (PCA) revealing the genes influencing differences among the study sites. Relative abundances were estimated using sample replicates (*n* = 2) for study sites (TL: The Lake in Central Park – New York, USA; RA: Raczyńskie Lake, Poland; SU: Sulejów Reservoir, Poland; SP: Singapore urban reservoir).

The relative abundance of selected nitrogen cycling genes and normalized abundances to the 16S rRNA of total bacteria (Supplementary Table S15) were used in a PCA (Figure 6), with coordinates 1 and 2 showing the highest variability for the discrimination of samples and replicates (up to 87.85%). Scores and correlations for the construction of PCA are described in Supplementary Table S16. Genes involved in N-degradation (*ure*B, *gln*A, *gcv*T), DNRA (*nap*A and *nar*GHZ) and ANRA (*nar*B) were detected across all sites. For TL, *ure*B, *gln*A, and *nar*B were most abundant (Figure 6a), which separated TL from the other sites (Figure 6b). At RA and SU, the highest abundances were of the N-fixing gene *nif*K (Figure 6a), which separated the Polish sites from the others (Figure 6b). At SP, the abundances of several unique genes (*nar*ZH, *nor*B, *nos*Z, *nxr*A, *nxr*B, and _c_*amo*A) were higher than the other sites (Figure 6a). Nitrifying genes were generally depleted in TL, RA, and SU (Figure 6a) compared to SP. ANNAMOX genes were not detected in any samples.

**Figure 7:**
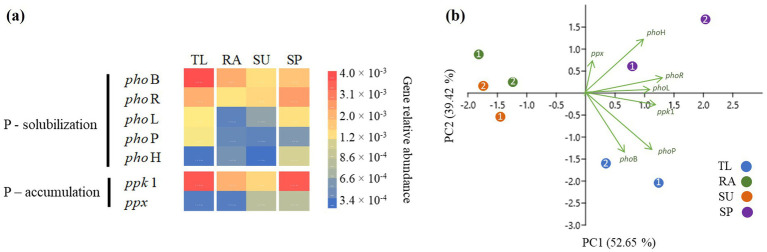
Relative abundances of phosphorus cycling genes in the study sites: **(a)** Heat map representing the differences in relative abundances of genes normalized with 16S rRNA of total bacteria; **(b)** principal components analysis (PCA) revealing the genes influencing differences among the study sites. Relative abundances were estimated using sample replicates (*n* = 2) for study sites (TL: The lake in Central Park – New York, USA; RA: Raczyńskie Lake, Poland; SU: Sulejów Reservoir, Poland; SP: Singapore urban reservoir).

The relative abundance of selected P-cycling genes and normalized abundances to the 16S rRNA of total bacteria (Supplementary Table S15) were used in a PCA (Figure 7) with coordinates 1 and 2, showing the highest variability for the discrimination of samples and replicates (up to 92.07%). Scores and correlations for the construction of PCA are described in Supplementary Table S16. Sites TL and SP were clearly more abundant in P-cycling genes (Figure 7a). Both sites had similar high abundances of P-solubilizing genes *pho*L and *pho*R, and the P-accumulating gene *ppk*1 (Figure 7a). TL had the highest abundances of *pho*B and *pho*P (Figure 7a), which differentiated TL from the other sites (Figure 7b). In contrast, SP had the highest abundance of *pho*H (Figure 7a), which was a factor in the differentiation of SP (Figure 7b). RA and SU had the lowest relative abundances of P-cycling genes (Figure 7a), clustering together separate at the opposite side of the PC1 axis (Figure 7b).

## 4 Discussion

The diversity and potential eco-physiology of global bacterioplankton communities during HCBs was investigated through synoptic sampling of four shallow freshwater sites located in three different continents. While TL and SP exhibited high P-PO_4_^3−^ concentrations, SU and RA had very low P-PO_4_^3−^ but measurable dissolved N-species (Table 1). Despite differences in nutrient conditions, all study lakes had phytoplankton communities dominated by cyanobacteria (35.6–68.7%; Figure 1a). However, the sites differed substantially in the cyanobacteria and associated bacterial community composition, toxigenicity, and eco-physiological functions.

It is important to acknowledge that this is a preliminary study based on a limited number of samples. While the dataset was sufficient for comparative analysis, results were interpreted with caution. Despite low sample sizes, the study provides baseline information and generates hypotheses about bacterial taxa potentially associated with cyanobacterial blooms. These findings provide a starting point for a broader, ongoing investigation with expanded sampling efforts across a wider range of freshwater systems.

### 4.1 Diversity of cyanobacteria and their toxigenicity

Colonial coccoidal forms of *Microcystis* were present in all sites, which is congruous with its ubiquitous nature and likely occurrence in eutrophic waters (Harke et al., 2016). At TL, *Microcystis* was dominant (Figure 1b) – an observation that was previously recorded annually from 2015 to 2020 (Flanzenbaum et al., 2022). The other three sites had higher abundances of colonial filamentous cyanobacteria (*Aphanizomenon* or *Planktothrix*; Figure 1b); in fact, RA and SU have had previously reported blooms composed of mixed assemblages (*Microcystis* and *Aphanizomenon*; Kowalczewska-Madura et al., 2022; Mankiewicz-Boczek and Font-Nájera, 2022). At SP, *Planktothrix* was dominant over *Microcystis*, which corresponds to previous observations that *Microcystis* blooms in this urban reservoir are being replaced by filamentous cyanobacteria (Te and Gin, 2011, and personal written communication of unpublished monitoring data by Te S. H. with Public Utilities Board of Singapore, 2024).

The cyanobacteria from the four sites had variable toxigenic potential (Figure 2a); however, all had the potential to produce MCs (Figure 2a). The strongest correlation between the *mcy*E gene and the abundance of *Microcystis* was at TL (Figure 2b). Flanzenbaum et al. (2022) corroborated elevated annual MCs and toxigenic potential at TL; they reported the highest gene copy numbers of *mcy*E occurred at mid- and post-summer seasons (up to 8.22 × 10^5^ copies mL^−1^) when *Microcystis* dominated. *Planktothrix* is also well known to produce MCs, but a significant negative correlation between the *mcy*E gene and this cyanobacterium was observed at SP. In contrast, *Microcystis* correlated with the *mcy*E gene indicating that this coccoidal cyanobacteria is responsible for the potential production of MCs at SP. RA had the highest toxigenic potential for CYN, ATX, and STX, which was associated with the highest diversity and relative abundances of cyanobacterial taxa (Figure 1b). Interestingly, *Aphanizomenon* is one of the cyanobacterial genera implicated in the production of CYN, ATX, and STX (Wang et al., 2021). However, the positive correlations observed between cyanotoxin genes and *Aphanizomenon* NIES81 were not significant after *p*-value Bonferroni correction in this study (Figure 2b). Despite this, the presence of diverse toxicity potential in filamentous forms—particularly when co-occurring with hepatotoxic *Microcystis* colonies—may pose a greater environmental threat than blooms dominated by a single cyanobacterial type. Additionally, filamentous cyanobacteria are known to produce a greater array of cyanotoxins that are known to have higher toxicity than MCs (Christensen and Khan, 2020). In another study, a mixed bloom of *Dolichospermum, Aphanizomenon, Planktothrix*, and *Microcystis* in Utah Lake (USA) revealed a similar toxigenic potential (MCs, CYN and ATX) using constructed metagenomes (Li et al., 2023), raising concerns about the usage of water resources. In 2018, restoration efforts to reduce the occurrence of HCBs have been implemented in RA including phosphorus inactivation and biomanipulation techniques that resulted in water quality improvements during that summer. However, reductions in these restoration efforts during the subsequent years contributed to water quality deterioration and HCB recurrence (Kowalczewska-Madura et al., 2022). In this study, the presence of mixed cyanobacterial taxa and strong potential toxigenicity for RA indicates that sustainable restoration efforts applied with the same intensity as in 2018 could reduce the occurrence of HBCs at RA.

### 4.2 Diversity and eco-physiological traits of associated bacteria

Bacterial communities play an important role in the development and decay of HCBs; therefore, knowledge of their association with particular cyanobacterial taxa could reveal potential eco-physiological roles that can exacerbate health threats. In this study, bacterial communities were most diverse in a *Microcystis*-dominated HCB (Figure 3). Extracellular polysaccharides substances (EPS), in the mucilage of *Microcystis* helped explain high bacterial diversity indices. Pannard et al. (2016) demonstrated that EPS production in *Microcystis aeruginosa* was significantly higher than that of the filamentous cyanobacteria, *Limnothrix* sp. and *Planktothrix agardhii* in controlled laboratory assays. High EPS production in coccoidal forms of cyanobacteria was attributed to higher cellular surface-to-volume ratios, allowing more area for bacterial attachment. Furthermore, higher carbon (C): N ratios in EPS of *M. aeruginosa* suggest a potential role as a C-rich substrate that could support heterotrophic bacterial communities. In the current study, the previously mentioned factors contribute to a deeper understanding of the reduced diversity of bacterial communities associated with HCBs dominated by the filamentous cyanobacterium *Planktothrix* (Figure 3). This knowledge can be further extended to blooms dominated by *Aphanizomenon*, a cyanobacterium for which EPS production has not specifically been compared to coccoidal forms of cyanobacteria.

Among the sites, the cyanosphere was composed of unique communities of bacterial taxa, depending on the dominant cyanobacteria. *Microcystis* blooms were interrelated with *Roseomonas* and the family Burkholderiaceae in TL and RA (Figure 4a)*. Roseomona*s contains genes involved in many metabolic pathways for obtaining energy from organic carbon (Cai et al., 2024), and Burkholderiaceae has numerous saprophytic strains known to utilize decaying organic matter (Coenye, 2014). Several other studies have described the interrelation of *Microcystis* with *Roseomonas* (Chun et al., 2020; Chen et al., 2020; Kim et al., 2019; Pérez-Carrascal et al., 2021) and Burkholderiaceae (Chun et al., 2020; Jankowiak and Gobler, 2020), although potential eco-physiological roles were not explicitly mentioned. Moreover, *Microcystis* was interrelated to Sphingobacteriales_env.OPS17 in TL (Figure 4a), another bacterial group described as *Microcystis* bloom specialists, favoring the uptake of cyanobacterial exudates and decaying material (Bagatini et al., 2014; Parulekar et al., 2017). The order Sphingobacteriales has been documented to contain microcystin-degrading bacteria (Kohler et al., 2014; Mankiewicz-Boczek and Font-Nájera, 2022), which was related to high MCs potential in *Microcystis* blooms (Figures 1b, 2a). These three bacterial taxa likely consist of opportunistic populations that thrive on the carbon-rich content of EPS within the *Microcystis* cyanosphere. Furthermore, it is probable that these three bacterial taxa play a substantial role in nutrient cycling processes within this environment. In the *Planktothrix* bloom in SP, a strong relation with *Rheinheimera* and *Flavobacterium* was observed (Figure 4a). *Rheinheimera* strains have been isolated from HCBs, and experimental laboratory assays have demonstrated their algicidal effects on *M. aeruginosa* (Wu et al., 2019), which suggests that this bacterium may regulate the growth of *Planktothrix* blooms in SP. *Flavobacterium* includes strains that are capable of cyanotoxin degradation and other recalcitrant and problematic organic compounds (Berg et al., 2009). Elevated dominance of *Flavobacterium* in *Planktothrix* blooms suggest that this bacterium plays a potential role in nutrient-cycling in the *Planktothrix* cyanosphere. Finally, in the case of *Aphanizomenon* (which was most dominant in SU), a strong relation was observed with *Paucibacter* (Figure 4a) – another potential algicidal bacteria shown to inhibit the growth of *Microcystis* and degrade cyanotoxins in a mesocosm study (Le et al., 2022b). *Paucibacter* has also been linked to a decline in cell growth of filamentous *Dolichospermum* blooms (Le et al., 2023), suggesting that it could be important for regulating *Aphanizomenon* blooms in SU. These results suggest that *Microcystis* blooms may have potentially stronger cyanobacterial-bacterial interactions, as indicated by the higher number of significant positive correlations observed between *Microcystis* and its attached bacterial consortia compared to *Planktothrix* or *Aphanizomenon* (Figure 5).

### 4.3 Potential pathogenic bacteria

HCBs could be sinks for dangerous pathogenic bacteria; however, 16S rRNA gene-based identification can only indicate the presence of genera with potential pathogenic members and cannot confirm pathogenicity at the strain level. Nevertheless, the cyanosphere has been described as an ideal environment for bacteria to confer resistance to many antimicrobial agents (Zhang et al., 2020). Knowledge about their presence within HCBs is essential because of additional threats to the health of ecosystems. In the present study, bacterial genera listed as priority pathogens by the World Health Organization (2024) – such as *Mycobacterium*, *Pseudomonas*, and *Acinetobacter* – were detected. However, their relative abundances were consistently low across all sites (<0.5% of relative abundances) and therefore, do not represent an elevated threat. Other bacteria that were frequently detected or associated with HCBs may pose a greater potential threat as pathogens. For example, *Roseomonas* was linked to *Microcystis* blooms in TL and RA (Figure 4a). Despite their ubiquity in natural environments, several strains have been isolated from clinical samples and are recognized as causes of opportunistic infections in humans (Ioannou et al., 2020). *Roseomonas* has been commonly associated with *Microcystis* blooms in previous studies (refer to section 4.2), although potential pathogenicity has not been properly addressed. In contrast, *Flavobacterium* may be an important potential pathogen for *Planktothrix* blooms in SP (Figure 4a). *Flavobacterium* is cosmopolitan in freshwater environments, nevertheless, most pathogenic strains have been isolated from animal tissue, and most cases of infection have been reported in freshwater fish (McBride, 2014). Zhang et al. (2020) reported the potential pathogenicity of *Flavobacterium* in *Planktothrix* blooms, and revealed that this bacterial group was associated with elevated amounts of antibiotic resistance genes. These observations underscore the need to further investigate the potential pathogenicity of *Roseomonas* and *Flavobacterium*, because they are key members of the consortia associated with coccoid and filamentous cyanobacterial blooms and appear to play important roles in nutrient cycling (refer to section 4.2).

### 4.4 Nutrient-transforming genes

The detection of nutrient-transforming bacterial genes varied significantly between sites dominated by non-diazotrophic *Microcystis* and *Planktothrix* (such as TL and SP, respectively) and those where diazotrophic *Aphanizomenon* was more abundant (RA and SU in Poland) (Figures 6, 7). In relation to N-cycling processes, the most abundant genes (*ure*B, *gln*A, *gcv*T, *nar*B, and *nar*ZHG) indicated a potential preference for ammonium utilization by phytoplankton communities dominated by cyanobacteria. At TL and SP, N levels were below detection limits (Table 1), suggesting rapid N uptake by non-diazotrophic cyanobacteria. Coping mechanisms for N-uptake in *Microcystis* blooms at TL were associated with high relative abundances of N-synthesis and N-degradation genes (*ure*B and *gln*A) and ANRA genes (*nar*B) (Figure 6). These genes are crucial to converting unavailable N to ammonium, which is largely regarded as the preferred N-species for *Microcystis* (Yan et al., 2023). In fact, these genes have been detected in other freshwater bodies with *Microcystis* blooms such as Lake Erie (OH, USA), Lake Agawam (NY, USA), and William H Harsha Lake (Lake Harsha, OH, USA) (Steffen et al., 2015; Gobler and Jankowiak, 2022; Wang et al., 2021). Wan et al. (2022) suggested that the N-degradation gene (*gln*A) and nitrate reduction gene (*nar*B) contributed to ammonium accumulation in Tangzun and Zhiyin Lakes (China), resulting from the decay of a *Dolichospermum* bloom. This accumulation likely facilitated the subsequent appearance of *Microcystis* in late summer. In the *Planktothrix* bloom in SP, the DNRA gene (*nar*HZ) was more abundant than other sites. Previous studies have suggested that *Microcystis* blooms harbor a large quantity of bacteria that perform DNRA, which is essential for N acquisition in non-diazotrophic cyanobacteria in N-limited environments (Yang et al., 2021; Yan et al., 2023; Wan et al., 2022; Cai et al., 2024). Observations in those studies indicated that *Planktothrix* might have similar N-scavenging strategies as have been previously demonstrated for *Microcystis*, especially considering that they both are non-diazotrophic. Furthermore, denitrifying genes (*nor*B and *nos*Z) and nitrifying genes (*nxr*A, *nxr*B and *_c_amo*A) were more abundant in *Planktothrix* blooms compared to *Microcystis* (Figure 6). Denitrifying and nitrifying bacteria may compete with *Microcystis* for nutrients, because they eliminate nitrogen in freshwater ecosystems. Similar observations have been described in previous studies, where nitrifying and denitrifying genes were generally depleted during a *Microcystis* bloom (Yang et al., 2021; Cai et al., 2024), indicating that *Planktothrix* may rely on different mechanisms for N obtainment that have not been identified or described. However, the dominance of *Planktothrix* in SP was not as pronounced as that of *Microcystis* in TL (Figure 1b). Consequently, it is possible that a greater number of N-cycling processes may have been observed because of reduced competitive pressures among bacterioplankton communities. In contrast, *Aphanizomenon* blooms at the Polish sites (RA and SU) were associated with the highest abundances of the N-fixing gene *nif*K (Figure 6) which probably contributed to the highest observed concentrations of N-species for those sites (up to 28.69 μg L^−1^ of N-NH_4_^+^ and 36.87 μg L^−1^ of N-NO_3_^−^, Table 1). Other studies have shown that metagenomes of *Aphanizomenon* and *Dolichospermum* blooms were comprised of N-fixing genes that were considered important biological factors supporting N-processes for other bacterioplankton (Li et al., 2023; Yang et al., 2021; Wan et al., 2022). In our study, N-fixing genes likely contributed to the sustained presence of mixed blooms of diazo- and non-diazotrophic cyanobacteria in RA and SU.

The non-diazotrophic taxa *Microcystis* and *Planktothrix* exhibited higher abundances of phosphorus cycling genes in their bacterial consortia. Alkaline phosphatases in particular, were abundant in *Microcystis* and *Planktothrix* blooms (*pho*BP and *pho*HR, respectively, Figure 7). Alkaline phosphatases are generally enriched in *Microcystis* blooms and have been associated with their ability to thrive in P-limited environments (Yang et al., 2021; Cai et al., 2024). However, both TL and SP exhibited high concentrations of phosphates (105.6 μg L^−1^ and 34.00 μg L^−1^ of P-PO_4_^3−^, respectively, Table 1) and were not P-limited. The shallowness and proximity to the sediments at these sites facilitates rapid microbial solubilization of organic phosphorus to inorganic forms (Flanzenbaum et al., 2022). Wan et al. (2022) suggests that the phenomenon may also be related to detritus from *Microcystis* decomposition, leading to phosphorus release and solubilization. While there are no specific observations for *Planktothrix* blooms, our results suggest a similar dynamic to that observed in *Microcystis*-dominated blooms. Furthermore, non-diazotrophic cyanobacteria were associated with high abundances of the *ppk*1 gene (Figure 6), highlighting their potential role in phosphorus accumulation and removal from water. However, the P-rich environments of TL and SP suggest that phosphorus is not a limiting factor for the growth of these cyanobacterial communities. In fact, both *Microcystis* and *Planktothrix* are known to thrive in P-rich waters (Kurmayer et al., 2016; Flanzenbaum et al., 2022; Wejnerowski et al., 2024). Finally, the low abundance of phosphorus-cycling genes associated with *Aphanizomenon* at RA and SU likely demonstrates the less complex microbial communities observed in their cyanosphere. However, additional studies would be needed to confirm this hypothesis because it was beyond the scope of our study.

Our study results underscore the importance of understanding the diversity of bacterial communities and genes associated with HCBs, as by revealing critical biological elements that enhance the persistence, toxicity and spread of HCBs in diverse ecosystems worldwide. This knowledge could help in elucidate the specific molecular mechanisms that enable cyanobacterial persistence in aquatic ecosystems, which could be used for developing more effective systemic solutions for the prevention, control and mitigation of HCBs. Our work represents an initial effort in the characterization of potential hazard elements associated with HCBs on a global scale, that could be extended to additional freshwater bodies in other countries in the near future.

## Data Availability

The datasets presented in this study can be found in online repositories. The names of the repository/repositories and accession number(s) can be found in the article/Materials and Methods section (Bioinformatic analyses).

## Footnotes

1
https://www.arb-silva.de/

2
https://www.ncbi.nlm.nih.gov/sra
