# Supplementary material for: Bacterial community diversity and potential eco-physiological roles in toxigenic blooms composed of Microcystis, Aphanizomenon or Planktothrix
Source: Front Microbiol. 2025 Dec 16;16:1655370. doi: 10.3389/fmicb.2025.1655370 (PMC12753108; doi:10.3389/fmicb.2025.1655370)
Supplement: Supplementary file 1 [file Data_Sheet_1.docx]

**Bacterial community diversity and potential eco-physiological roles in toxigenic blooms composed of *Microcystis*, *Aphanizomenon* or *Planktothrix***

Joanna Mankiewicz‑Boczek^1*^, Arnoldo Font‑Nájera^2**^, Karina Yew-Hoong Gin^3^, Jennifer L. Graham^4^, Dominik Strapagiel^5^, Rebecca M. Gorney^4^, Jerome Wai Kit Kok^3^, Shu Harn Te^3^, Magdalena Kluska^2^, Milena Skóra^5^, Michał Seweryn^5^, Francisco Lopez-Hun^6,1^

^1^University of Lodz, Faculty of Biology and Environmental Protection, UNESCO Chair on Ecohydrology and Applied Ecology, Lodz, Poland

^2^European Regional Centre for Ecohydrology of the Polish Academy of Sciences, Tylna 3, 90-364, Lodz, Poland

^3^Department of Civil and Environmental Engineering, National University of Singapore, Singapore

^4^U.S. Geological Survey, Troy, New York, USA

^5^University of Lodz, Faculty of Biology and Environmental Protection; Biobank Laboratory, Lodz, Poland

^6^University of Lodz and institutes of the Polish Academy of Sciences, Doctoral School BioMedChem, Lodz, Poland.

Joanna Mankiewicz‑Boczek [joanna.mankiewicz@biol.uni.lodz.pl](mailto:joanna.mankiewicz@biol.uni.lodz.pl) (corresponding author *)

Arnoldo Font‑Nájera [a.font-najera@erce.unesco.lodz.pl](mailto:a.font-najera@erce.unesco.lodz.pl) (corresponding author **)

Karina Yew-Hoong Gin [ceeginyh@nus.edu.sg](mailto:ceeginyh@nus.edu.sg)

Jennifer L. Graham [jlgraham@usgs.gov](mailto:jlgraham@usgs.gov)

Dominik Strapagiel [dominik.strapagiel@biol.uni.lodz.pl](mailto:dominik.strapagiel@biol.uni.lodz.pl)

Rebecca M. Gorney [rgorney@usgs.gov](mailto:rgorney@usgs.gov)

Jerome Wai Kit Kok [jerome.kok@nus.edu.sg](mailto:jerome.kok@nus.edu.sg)

Shu Harn Te [eritsh@nus.edu.sg](mailto:eritsh@nus.edu.sg)

Magdalena Kluska m.kluska@erce.unesco.lodz.pl

Milena Skóra [milena.skora@biol.uni.lodz.pl](mailto:milena.skora@biol.uni.lodz.pl)

Michał Seweryn [michal.seweryn@biol.uni.lodz.pl](mailto:michal.seweryn@biol.uni.lodz.pl)

Francisco Lopez-Hun [francisco.lopez.hun@edu.uni.lodz.pl](mailto:francisco.lopez.hun@edu.uni.lodz.pl)

**Supplementary Material**

**
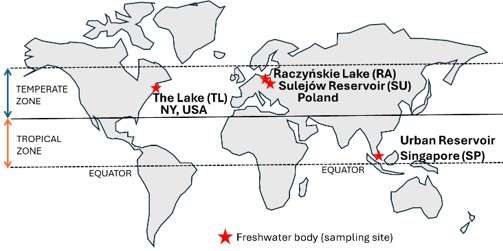
**

**Fig S1.** Location of study sites (TL: The Lake in Central Park (U.S. Geological Survey station 404630073580801; U.S. Geological Survey, 2025)– New York, USA; RA: Raczyńskie Lake, Poland; SU: Sulejów Reservoir, Poland; SP: Singapore urban reservoir) at a global scale.


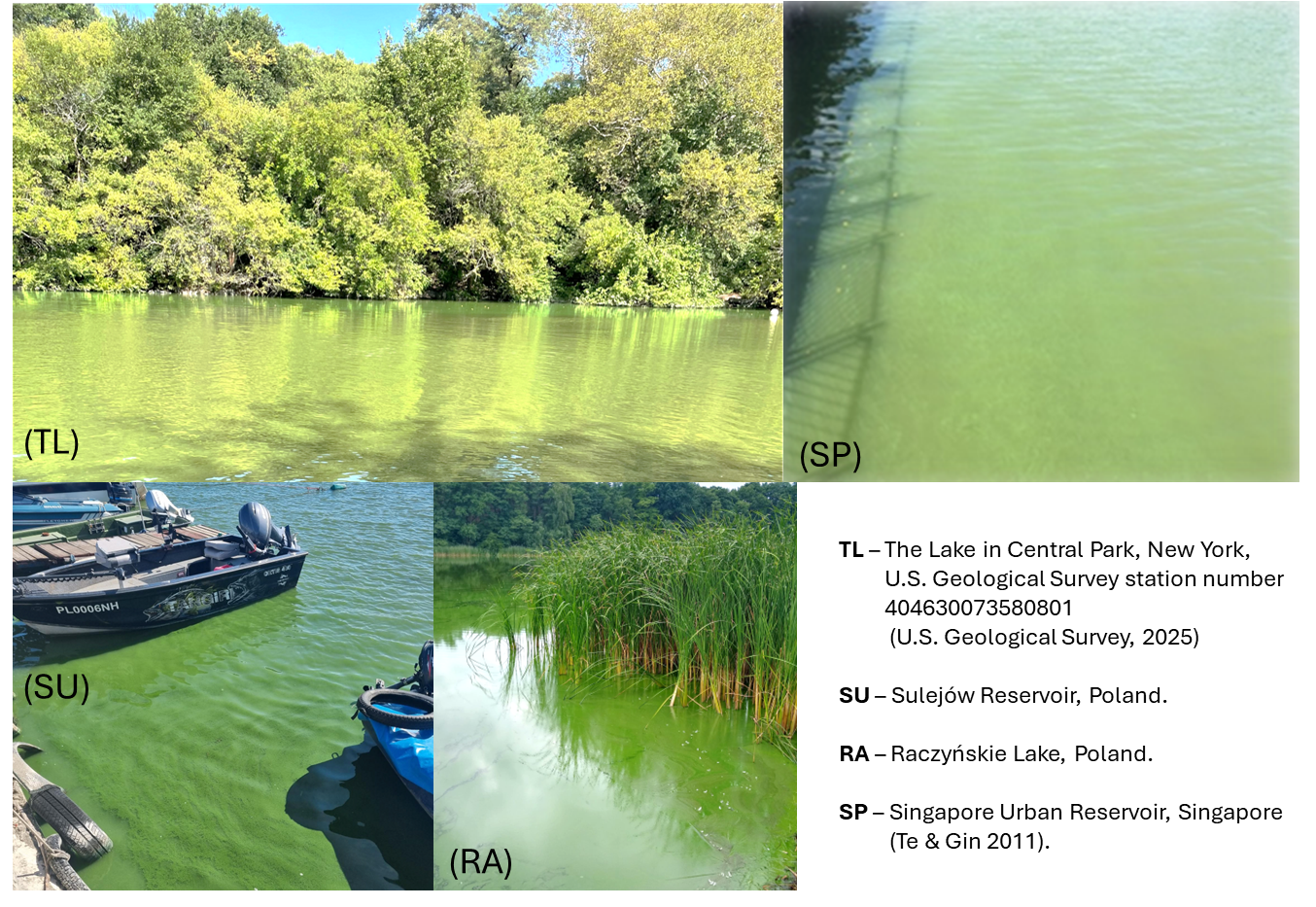


**Fig S2**. Field sampling collection during visible cyanobacterial bloom formations. TL by Rebecca Gorney; SU by Joanna Mankiewicz-Boczek; RA by Arnoldo Font-Nájera; SP by Shu-Harn Te.

**Table S1**. Summary of genes mined in shotgun metagenomes.

| **Gene** | **Accession number** | **Name of the accession** | **Size of the published sequence (bp)** | **Function** | **Category** |  |
| --- | --- | --- | --- | --- | --- | --- |
| ***gln*A** | AF027612.1 | *Synechococcus* WH5701 glutamine synthetase (glnA) gene, partial cds | 746 | Ammonia assimilation – Nitrogen decomposition | N-cycling |  |
| ***ure*B** | KP401975 | *Helicobacter* *pylori* strain RCGLD HC715 UreB (ureB) gene, partial cds | 1082 | Ammonia assimilation – Nitrogen decomposition | N-cycling |  |
| ***gcv*T** | DQ362419.1 | *Escherichia* *coli* strain 1708 GcvT (gcvT) gene, partial cds | 416 | Ammonia assimilation – Nitrogen decomposition | N-cycling |  |
| ***glt*B** | JN049713.1 | *Burkholderia* sp. SD2570B GltB (gltB) gene, partial cds | 600 | ammonium assimilation - glutamate metabolism | N-cycling |  |
| ***glt*D** | CP001154.1:698686-700098 | GltD [*Laribacter* *hongkongensis* HLHK9] | 1412 | Ammonia assimilation – Nitrogen decomposition | N-cycling |  |
| ***amt*** | CP001087.1:5129340-5130653 | Amt [*Desulforapulum autotrophicum* HRM2] | 1314 | Ammonia assimilation – Nitrogen decomposition | N-cycling |  |
| ***amo*A** | EF175100.1 | *Nitrosospira* sp. EnI299 AmoA (amoA) gene, partial cds | 669 | ammonium oxidation (nitrification) | N-cycling |  |
| ***nxr*A** | MW910243.1 | Uncultured *Nitrobacter* sp. NxrA gene, partial cds | 327 | nitrite oxidation (nitrification) | N-cycling |  |
| ***nxr*B** | MZ614340.1 | Uncultured *Nitrospira* sp. clone nxrB-1 nitrite oxidoreductase subunit B (nxrB) gene, partial cds | 485 | nitrite oxidation (nitrification) | N-cycling |  |
| **_c_*amo*A** | MW023057.1 | Uncultured bacterium clone PIE49-H4 comammox ammonia monooxygenase (amoA) gene, partial cds | 406 | complete ammonium oxidation (COMAMMOX) | N-cycling |  |
| ***hzs*A** | JN703685.1 | Anammox bacterium enrichment culture clone cc2B_139d hydrazine synthase subunit A (hzsA) gene, partial cds | 1293 | anaerobic ammonia oxidation (ANAMMOX) | N-cycling |  |
| ***hzo*** | JQ901837.1 | Uncultured anaerobic ammonium-oxidizing bacterium clone 48H-H-19 HZO gene, partial cds | 994 | anaerobic ammonia oxidation (ANAMMOX) | N-cycling |  |
| ***nap*A** | KY316375.1 | *Pseudomonas* sp. strain HS-2 NapA (napA) gene, partial cds | 861 | Nitrate reduction (ANRA / DNRA) | N-cycling |  |
| ***nar*G** | MF627183.1 | Uncultured bacterium clone N.152 NarG (narG) gene, partial cds | 654 | Nitrate reduction (ANRA / DNRA) | N-cycling |  |
| ***nar*B** | FJ425281.1 | Uncultured bacterium clone HT4015Ne assimilatory nitrate reductase (narB) gene, partial cds | 591 | Nitrate reduction (ANRA / DNRA) | N-cycling |  |
| ***nar*Z** | CP006584.1:1321937-1325680 | *Escherichia coli* LY180, complete genome; nitrate reductase complete gene | 3744 | Nitrate reduction (ANRA / DNRA) | N-cycling |  |
| ***nar*H** | AJ288134.1 | *Pseudomonas* sp. partial narH gene for nitrate reductase beta-subunit isolate Ki-1z | 983 | Nitrate reduction (ANRA / DNRA) | N-cycling |  |
| ***nrf*A** | EF414596.1 | Uncultured *Geobacter* sp. clone BEMNRFA NrfA (nrfA) gene, partial cds. | 733 | Dissimilatory nitrate reduction to ammonium (DNRA) | N-cycling |  |
| ***nrf*H** | CP042170.1:2374000-2374620 | *Flavobacterium* sp. KBS0721 chromosome, complete genome; nitrite reductase gene | 621 | Dissimilatory nitrate reduction to ammonium (DNRA) | N-cycling |  |
| ***nir*B** | EF191163.1 | *Salmonella choleraesuis* strain 79500 NirB (nirB) gene, promoter region and partial cds | 738 | Denitrification | N-cycling |  |
| ***nir*K** | KX598844.1 | Uncultured bacterium clone S4S_nirK_6 NirK (nirK) gene, partial cds | 515 | Denitrification | N-cycling |  |
| ***nor*B** | L76189.1 | *Nitrobacter winogradskyi* NorB (norB) gene, partial cds | 1033 | Denitrification | N-cycling |  |
| ***nor*R** | AY266422.1 | *Staphylococcus aureus* subsp. aureus NCTC 8325 NorR (norR) gene, complete cds | 851 | Denitrification | N-cycling |  |
| ***nos*Z** | AB809000.1 | Uncultured *Pseudomonas* sp. nosZ gene for nitrous oxide reductase, partial cds, isolate: NosZEc.26 | 477 | Denitrification | N-cycling |  |
| ***nif*B** | JN600444.1 | *Pseudomonas* putida strain MB-L NifB (nifB) gene, partial cds | 1461 | N fixation | N-cycling |  |
| ***nif*K** | KU886165.1 | *Nostoc flagelliforme* NX-09 NifK (nifK) gene, complete cds | 1536 | N fixation | N-cycling |  |
| ***pho*B** | X81000.1 | *Shigella flexneri* PhoB gene | 1320 | Phosphorus solubilization | P-cycling |  |
| ***pho*B1** | FO082820.1:1216716-1217399 | response regulator PhoB1 in two-component regulatory system with PhoR (or CreC), regulation of Pi uptake (OmpR family) [*Pseudorhizobium banfieldiae*] | 684 | Phosphorus solubilization | P-cycling |  |
| ***pho*D** | XM_024716776 | *Plasmopara halstedii* phoD (PHALS_02303), partial mRNA | 1305 | Phosphorus solubilization | P-cycling |  |
| ***pho*H** | AF035751.1:3941-4485 | PhoH, partial [*Picosynechococcus* sp. PCC 7002] | 545 | Phosphorus solubilization | P-cycling |  |
| ***pho*L** | UIRQ01000001.1:781836-783227 | phosphate starvation-inducible protein (PhoH-like) [*Acinetobacter baumannii*] | 1392 | Phosphorus solubilization | P-cycling |  |
| ***pho*P** | MT052013.1 | *Klebsiella pneumoniae* strain k86 PhoP (phoP) gene, complete cds | 672 | Phosphorus solubilization | P-cycling |  |
| ***ppk*1** | LC079032.1 | *Rhodococcus erythropolis* ppk1 gene for polyphosphate kinase 1, partial cds, a part of 5' portion, strain: N9T-4 | 1226 | Polyphosphate accumulation | P-cycling |  |
| ***ppx*** | AF053463.1:682-2202 | exopolyphosphatase [*Pseudomonas aeruginosa*] | 1521 | Polyphosphate dissociation | P-cycling |  |
| ***mcy*E** | MF947374.1 | Uncultured cyanobacterium clone *Bergknappweiher*_0809_12 McyE (mcyE) gene, partial cds | 758 | Synthesis of microcystins | Cyanotoxin synthesis |  |
| ***ana*C** | KM245024.1:12215-17833 | *Cuspidothrix issatschenkoi* CHABD3 anatoxin-a synthetase gene cluster, complete sequence | 581 | Synthesis of anatoxin | Cyanotoxin synthesis |  |
| ***cyr*J** | ACYA01000027.1:C95408-94629 | *Cylindrospermopsis raciborskii* CS-505 CS505_3, whole genome shotgun sequence, CYRJ gene. | 690 | Synthesis of cylindrospermopsin | Cyanotoxin synthesis |  |
| ***sxt*A** | LT549449.1:c7745-4041 | *Aphanizomenon gracile* NIVA-CYA 676 genome sequence, strain NIVA-CYA 676, SXTA gene. | 684 | Synthesis of saxitoxin | Cyanotoxin synthesis |  |
|  |  |  |  |  |  |  |

**Table S2.** Description of diversity indices utilized to describe bacterial communities using 16S rRNA amplicons.

| **Index description** |
| --- |
| **Simpsons dominance (*D*)**  Ranges from 0 (all taxa are equally present) to 1 (one taxon dominates the community completely).  $D= \sum_{i} \left( \frac{ni}{n} \right)^{2}$  Where *ni* is the number of individuals of taxon i. |
| **Shannon diversity (*H’*)**  A diversity index, taking into account the number of individuals as well as number of taxa. Varies from 0 for communities with only a single taxon to high values for communities with many taxa, each with few individuals  $H= -\sum_{i} \frac{ni}{n}ln\frac{ni}{n}$  Where *ni* is the number of individuals of taxon i. |
| **Equitability (J)**  Shannon diversity divided by the logarithm of number of taxa. This measures the evenness with which individuals are divided among the taxa present. |
| **abundance-based coverage richness (ACE)**  An estimate of total species richness.  ACE = S + F1(F1 - 1) / (2 (F2 + 1))  where F1 is the number of singleton species and F2 the number of doubleton species. |

Indices were estimated with the specifications of the PAST program (version 4.12b) – detailed description can be obtained from online manual (<https://academia.edu/5978612/Past_manual>). Original description of indices was obtained from Harper (1999).

**Table S3.** Summary of track reads during the filtering process of 16S rRNA amplicons

| **Sample - replicate** | **input** | **filtered** | **denoisedF** | **denoisedR** | **merged** | **nochim** |
| --- | --- | --- | --- | --- | --- | --- |
| **TL-1** | 249832 | 208755 | 205675 | 205940 | 199117 | 197762 |
| **TL-2** | 304418 | 256233 | 251770 | 252389 | 240753 | 237667 |
| **TL-3** | 269114 | 226185 | 222639 | 222708 | 214604 | 212733 |
| **RA-1** | 320713 | 267388 | 264360 | 264786 | 256795 | 240852 |
| **RA-2** | 277762 | 231234 | 228840 | 228955 | 222221 | 209117 |
| **RA-3** | 300744 | 252211 | 249314 | 249677 | 241208 | 223243 |
| **SU-1** | 268729 | 216413 | 214247 | 214602 | 208254 | 204306 |
| **SU-2** | 245992 | 197914 | 195702 | 196297 | 189878 | 186563 |
| **SU-3** | 286167 | 236084 | 233667 | 233855 | 225383 | 219398 |
| **SP-1** | 420416 | 357457 | 353456 | 354205 | 336933 | 312514 |
| **SP-2** | 283370 | 235359 | 232498 | 232782 | 221647 | 209440 |
| **SP-3** | 285621 | 239731 | 236753 | 237167 | 224062 | 207842 |
| **TOTAL** | 3512878 | 2924964 | 2888921 | 2893363 | 2780855 | 2661437 |

From an input of 3,512,878 sequences, a total of 2,780,855 sequences were recovered as good quality sequences after filtration, denoising and merging with the pipeline DADA2 1.32 (Callahan et al., 2016) in R environment 4.3.2 (R core Team 2023). A total of 2,661,437 sequences were good for bacterial classification after chimera removal (nochim). TL: The Lake in Central Park – New York, USA; RA: Raczyńskie Lake, Poland; SU: Sulejów Reservoir, Poland; SP: Singapore urban reservoir.

**
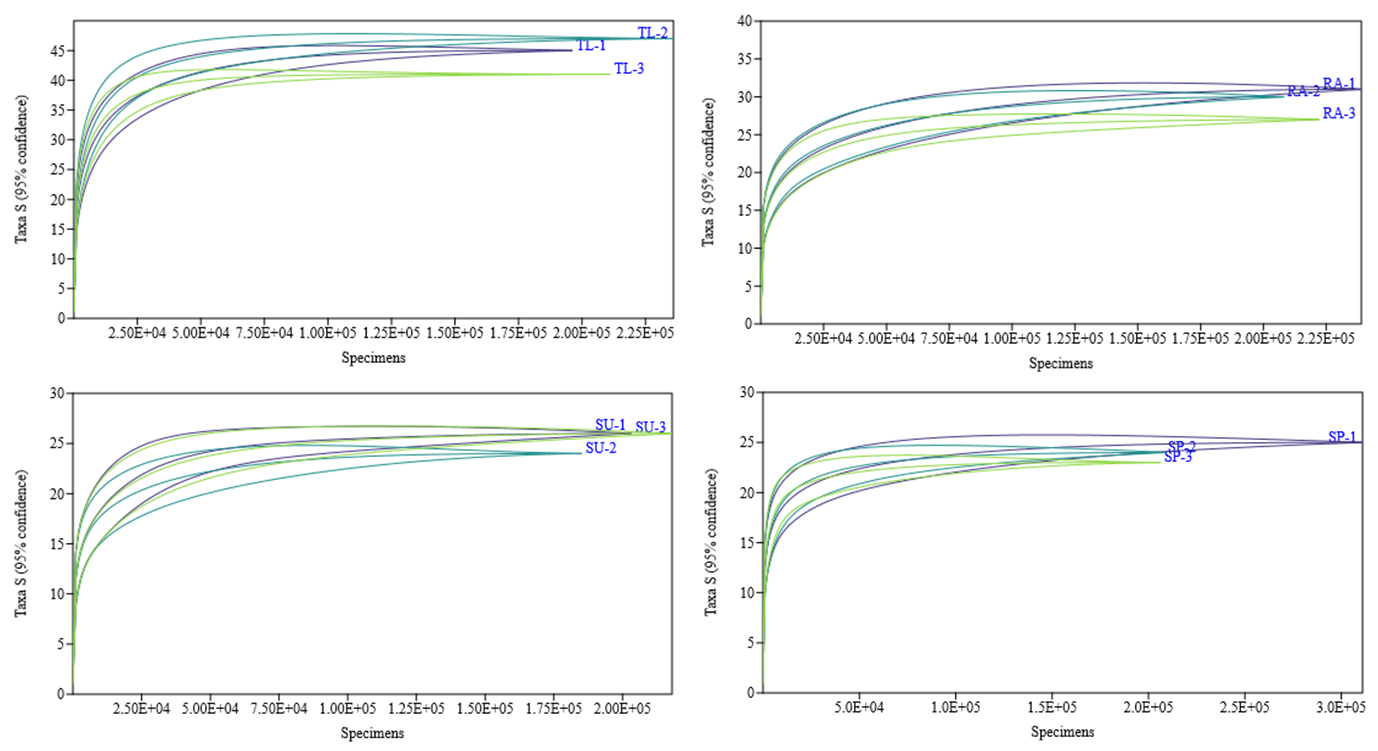
**

**Fig S3**. Individual rarefaction curves estimating the number of expected taxa according to the size of the sample (phylum level). Specimens refer to the number of sequences. TL: The Lake in Central Park – New York, USA; RA: Raczyńskie Lake, Poland; SU: Sulejów Reservoir, Poland; SP: Singapore urban reservoir. Numbers 1-3 represent the replicates.

**Table S4**. Number of total reads of 16S rRNA classified to bacterial phyla.

| **Taxa** | **TL** | | | | |  | **RA** | | | | |
| --- | --- | --- | --- | --- | --- | --- | --- | --- | --- | --- | --- |
|  | **1** | **2** | **3** | **Average** | **(%)** |  | **1** | **2** | **3** | **Average** | **(%)** |
| Cyanobacteria | 84088 | 83172 | 83915 | 27908.3 | 38.8 |  | 162006 | 142057 | 158474 | 51393.0 | 68.7 |
| Proteobacteria | 42383 | 56154 | 46094 | 16070.1 | 22.3 |  | 37108 | 32991 | 33598 | 11521.9 | 15.4 |
| Bacteroidetes | 37129 | 50543 | 42908 | 14508.9 | 20.1 |  | 12975 | 9623 | 7843 | 3382.3 | 4.5 |
| Verrucomicrobia | 8791 | 12200 | 9170 | 3351.2 | 4.7 |  | 7973 | 6046 | 5243 | 2140.2 | 2.9 |
| Actinobacteria | 11340 | 14726 | 14873 | 4548.8 | 6.3 |  | 2642 | 2227 | 2070 | 771.0 | 1.0 |
| Chloroflexi | 3846 | 5713 | 4974 | 1614.8 | 2.2 |  | 11596 | 10816 | 11043 | 3717.2 | 5.0 |
| Planctomycetes | 2302 | 3792 | 1898 | 888.0 | 1.2 |  | 2491 | 1890 | 1688 | 674.3 | 0.9 |
| Firmicutes | 362 | 528 | 351 | 137.9 | 0.2 |  | 1104 | 948 | 952 | 333.8 | 0.4 |
| Acidobacteria | 1239 | 1744 | 1688 | 519.0 | 0.7 |  | 1104 | 982 | 965 | 339.0 | 0.5 |
| Gemmatimonadetes | 3011 | 4330 | 2726 | 1118.6 | 1.6 |  | 240 | 173 | 191 | 67.1 | 0.1 |
| Others | 3271 | 4765 | 4136 | 1352.4 | 1.9 |  | 1613 | 1364 | 1176 | 461.4 | 0.6 |
| **Total** | 197762 | 237667 | 212733 | 72018.0 | 100.0 |  | 240852 | 209117 | 223243 | 74801.3 | 100.0 |
|  |  |  |  |  |  |  |  |  |  |  |  |
| **Taxa** | **SU** | | | | |  | **SP** | | | | |
|  | **1** | **2** | **3** | **Average** | **(%)** |  | **1** | **2** | **3** | **Average** | **(%)** |
| Cyanobacteria | 107499 | 95494 | 113648 | 35182.3 | 51.9 |  | 113866 | 78867 | 66775 | 28834.2 | 35.6 |
| Proteobacteria | 41522 | 37657 | 46030 | 13912.1 | 20.5 |  | 72533 | 49953 | 55603 | 19787.7 | 24.4 |
| Bacteroidetes | 44867 | 44618 | 48396 | 15320.1 | 22.6 |  | 99077 | 61341 | 68663 | 25453.4 | 31.4 |
| Verrucomicrobia | 2154 | 1906 | 2422 | 720.2 | 1.1 |  | 6158 | 3412 | 4316 | 1542.9 | 1.9 |
| Actinobacteria | 3177 | 2729 | 3325 | 1025.7 | 1.5 |  | 5260 | 5050 | 3267 | 1508.6 | 1.9 |
| Chloroflexi | 184 | 134 | 201 | 57.7 | 0.1 |  | 8867 | 6082 | 5325 | 2252.7 | 2.8 |
| Planctomycetes | 911 | 822 | 1197 | 325.6 | 0.5 |  | 3163 | 1975 | 1722 | 762.2 | 0.9 |
| Firmicutes | 2271 | 1697 | 2158 | 680.7 | 1.0 |  | 1119 | 863 | 404 | 265.1 | 0.3 |
| Acidobacteria | 844 | 690 | 1005 | 282.1 | 0.4 |  | 608 | 600 | 427 | 181.7 | 0.2 |
| Gemmatimonadetes | 326 | 367 | 457 | 127.8 | 0.2 |  | 105 | 41 | 64 | 23.3 | 0.0 |
| Others | 551 | 449 | 559 | 173.2 | 0.3 |  | 1758 | 1256 | 1276 | 476.7 | 0.6 |
| Total | 204306 | 186563 | 219398 | 67807.4 | 100.0 |  | 312514 | 209440 | 207842 | 81088.4 | 100.0 |

TL: The Lake in Central Park – New York, USA; RA: Raczyńskie Lake, Poland; SU: Sulejów Reservoir, Poland; SP: Singapore urban reservoir. Numbers 1-3 represent the replicates.

**Table S5**. Number of total reads of 16S rRNA classified to cyanobacteria strains.

| Taxa | **TL** | | | | |  | **RA** | | | | |
| --- | --- | --- | --- | --- | --- | --- | --- | --- | --- | --- | --- |
|  | **1** | **2** | **3** | **Average** | **(%)** |  | **1** | **2** | **3** | **Average** | **(%)** |
| *Microcystis*_PCC-7914 | 72868 | 65234 | 70119 | 69407.0 | 33.4 |  | 53822 | 45953 | 50734 | 50169.7 | 22.8 |
| *Planktothrix*_NIVA-CYA_15 | 1522 | 2258 | 2250 | 2010.0 | 1.0 |  | 12701 | 10537 | 12477 | 11905.0 | 5.4 |
| *Aphanizomenon*_MDT14a | 0 | 14 | 0 | 4.7 | 0.0 |  | 0 | 2 | 0 | 0.7 | 0.0 |
| *Snowella*_0TU37S04 | 0 | 13 | 10 | 7.7 | 0.0 |  | 25749 | 25625 | 26967 | 26113.7 | 11.9 |
| *Aphanizomenon*_NIES81 | 0 | 0 | 0 | 0.0 | 0.0 |  | 44025 | 37853 | 42961 | 41613.0 | 18.9 |
| *Cyanobium*_PCC-6307 | 2943 | 4996 | 1465 | 3134.7 | 1.5 |  | 462 | 372 | 480 | 438.0 | 0.2 |
| *Pseudanabaena*_PCC-7429 | 0 | 0 | 0 | 0.0 | 0.0 |  | 6170 | 5307 | 6015 | 5830.7 | 2.6 |
| *Dolichospermum*_NIES41 | 0 | 0 | 0 | 0.0 | 0.0 |  | 4910 | 4013 | 4360 | 4427.7 | 2.0 |
| *Cuspidothrix*_LMECYA_163 | 719 | 1290 | 944 | 984.3 | 0.5 |  | 2761 | 2377 | 3095 | 2744.3 | 1.2 |
| Other bacteria | 113812 | 154696 | 128996 | 132501.3 | 63.7 |  | 85559 | 73310 | 71713 | 76860.7 | 34.9 |
| total | 191864 | 228501 | 203784 | 208049.7 | 100.0 |  | 236159 | 205349 | 218802 | 220103.3 | 100.0 |
|  |  |  |  |  |  |  |  |  |  |  |  |
| Taxa | **SU** | | | | |  | **SP** | | | | |
|  | **1** | **2** | **3** | **Average** | **(%)** |  | **1** | **2** | **3** | **Average** | **(%)** |
| *Microcystis*_PCC-7914 | 14968 | 13727 | 16065 | 14920.0 | 7.5 |  | 11578 | 6097 | 7531 | 8402.0 | 3.6 |
| *Planktothrix*_NIVA-CYA_15 | 775 | 558 | 718 | 683.7 | 0.3 |  | 48109 | 41382 | 33007 | 40832.7 | 17.6 |
| *Aphanizomenon*_MDT14a | 77779 | 68932 | 81539 | 76083.3 | 38.2 |  | 0 | 0 | 5 | 1.7 | 0.0 |
| *Snowella*_0TU37S04 | 2990 | 2488 | 3023 | 2833.7 | 1.4 |  | 15263 | 8806 | 5548 | 9872.3 | 4.2 |
| *Aphanizomenon*_NIES81 | 0 | 9 | 0 | 3.0 | 0.0 |  | 7 | 0 | 0 | 2.3 | 0.0 |
| *Cyanobium*_PCC-6307 | 197 | 158 | 168 | 174.3 | 0.1 |  | 8858 | 6365 | 4034 | 6419.0 | 2.8 |
| *Pseudanabaena*_PCC-7429 | 380 | 242 | 324 | 315.3 | 0.2 |  | 482 | 338 | 240 | 353.3 | 0.2 |
| *Dolichospermum*_NIES41 | 1991 | 1507 | 1712 | 1736.7 | 0.9 |  | 17 | 15 | 10 | 14.0 | 0.0 |
| *Cuspidothrix*_LMECYA_163 | 1912 | 1332 | 1726 | 1656.7 | 0.8 |  | 0 | 0 | 0 | 0.0 | 0.0 |
| Other bacteria | 98906 | 93573 | 109628 | 100702.3 | 50.6 |  | 212617 | 138971 | 147776 | 166454.7 | 71.6 |
| Total | 199898 | 182526 | 214903 | 199109.0 | 100.0 |  | 296931 | 201974 | 198151 | 232352.0 | 100.0 |

TL: The Lake in Central Park – New York, USA; RA: Raczyńskie Lake, Poland; SU: Sulejów Reservoir, Poland; SP: Singapore urban reservoir. Numbers 1-3 represent the replicates. Mitochondrial and Chloroplast sequences were removed from the total number of sequences for each replicate.

**Table S6**. Editing features and statistic error for shotgun reads (metagenomes).

|  | TrimmomaticPE | | | Vsearch | | | | | | | | | |
| --- | --- | --- | --- | --- | --- | --- | --- | --- | --- | --- | --- | --- | --- |
| **Sample replicate** | Total reads | Input pairs | Good quality pairs | **Merged reads** | | **Statistics of merged reads** | | | | | | | |
|  |  |  |  |  |  | Mean read length (bp) | Standard deviation of fragment length | Mean expected errors in: | | | Mean observed errors in: | | |
|  |  |  |  | Total number | % |  |  | forward sequences | reverse sequences | merged sequences | merged region of forward sequences | merged region of reverse sequence | merged region |
| TL_1 | 25392427 | 17995555 | 16990336 | 8588245 | 50.5 | 340.09 | 72.75 | 0.74 | 0.78 | 0.52 | 0.57 | 0.55 | 1.23 |
| TL_2 | 22909970 | 14187551 | 13699736 | 4489502 | 32.8 | 323.71 | 77.64 | 0.71 | 0.69 | 0.43 | 0.56 | 0.48 | 1.03 |
| RA_1 | 22547522 | 15766380 | 14990212 | 7432902 | 49.6 | 336.38 | 73.2 | 0.65 | 0.69 | 0.44 | 0.48 | 0.47 | 0.95 |
| RA_2 | 44980993 | 29419444 | 28304584 | 11628175 | 41.4 | 324.83 | 76.01 | 0.66 | 0.69 | 0.42 | 0.48 | 0.48 | 0.96 |
| SU_1 | 25039315 | 17694822 | 16876800 | 8714285 | 51.6 | 339.07 | 72.33 | 0.67 | 0.69 | 0.45 | 0.49 | 0.47 | 0.96 |
| SU_2 | 35684103 | 22784689 | 21997813 | 8311523 | 37.8 | 324.09 | 76.18 | 0.66 | 0.65 | 0.4 | 0.49 | 0.44 | 0.93 |
| SP_1 | 12918602 | 8780682 | 8310997 | 3703392 | 44.6 | 338.53 | 75.08 | 0.65 | 0.67 | 0.45 | 0.49 | 0.45 | 0.94 |
| SP_2 | 22226849 | 13862149 | 13253788 | 4280727 | 32.3 | 331.93 | 79.39 | 0.65 | 0.68 | 0.44 | 0.48 | 0.46 | 0.94 |
| average | 26462473 | 17561409 | 16803033.3 | 7143593.875 | 42.575 | 332.32875 | 75.3225 | 0.67375 | 0.6925 | 0.44375 | 0.505 | 0.475 | 0.9925 |

Pairs that failed merging were due to any of the following: i) few chimeras found on same diagonal; ii) multiple potential alignments; iii) too many differences, iv) alignment score too low, or score drop too high; v) overlap too short; vi) staggered read pairs. TL: The Lake in Central Park – New York, USA; RA: Raczyńskie Lake, Poland; SU: Sulejów Reservoir, Poland; SP: Singapore urban reservoir.

**Table S7**. Composition of selected cyanotoxin genes in metagenomic shotguns.

| **Genes** | **TL_1** | | **TL_2** | | **RA_1** | | **RA_2** | | **SU_1** | | **SU_2** | | **SI_1** | | **SI_2** | |
| --- | --- | --- | --- | --- | --- | --- | --- | --- | --- | --- | --- | --- | --- | --- | --- | --- |
|  | HN | Normalized | HN | Normalized | HN | Normalized | HN | Normalized | HN | Normalized | HN | Normalized | HN | Normalized | HN | Normalized |
| *mcy*E | 704 | 3.91E-02 | 639 | 4.54E-02 | 53 | 4.46E-03 | 90 | 7.15E-03 | 95 | 2.72E-03 | 190 | 2.51E-03 | 18 | 1.16E-03 | 15 | 6.54E-04 |
| *ana*C | 5 | 2.77E-04 | 1 | 7.10E-05 | 30 | 1.54E-03 | 48 | 1.34E-03 | 3 | 1.41E-04 | 3 | 1.13E-04 | 5 | 3.23E-04 | 3 | 1.31E-04 |
| *cyr*J | 1 | 5.55E-05 | 0 | 0.00E+00 | 23 | 1.18E-03 | 44 | 1.23E-03 | 1 | 4.70E-05 | 1 | 3.77E-05 | 8 | 5.18E-04 | 0 | 0.00E+00 |
| *sxt*A | 2 | 1.11E-04 | 0 | 0.00E+00 | 4 | 2.05E-04 | 11 | 3.06E-04 | 2 | 9.39E-05 | 1 | 3.77E-05 | 0 | 0.00E+00 | 0 | 0.00E+00 |
| 16S rRNA | 18023 | | 14081 | | 19505 | | 35896 | | 21298 | | 26555 | | 15458 | | 22931 | |

HN: hit numbers; normalized to the total number of 16S rRNA gene. TL: The Lake in Central Park – New York, USA; RA: Raczyńskie Lake, Poland; SU: Sulejów Reservoir, Poland; SP: Singapore urban reservoir.

**Table S8**. Spearman’s correlations between the relative abundance of cyanobacterial strains and the normalized cyanotoxin synthetase gene counts (shotguns).

| **Cyanobacterial taxon** | ***mcy*E** | | ***ana*C** | | ***cyr*J** | | ***sxt*A** | |
| --- | --- | --- | --- | --- | --- | --- | --- | --- |
|  | *r* | *p* | *r* | *p* | *r* | *p* | *r* | *p* |
| *Microcystis*_PCC-7914 | **0.95238** | **0.0011409 (0.04739)** | 0.11905 | 0.79301 (1) | 0.21557 | 0.60645 (1) | 0.48795 | 0.22708 (1) |
| *Planktothrix*_NIVA-CYA_15 | -0.40476 | 0.32684 (1) | 0.33333 | 0.40863 (1) | 0.11976 | 0.77927 (1) | -0.26837 | 0.52768 (1) |
| *Aphanizomenon*_MDT14a | 0.027277 | 0.97619 (1) | -0.68193 | 0.089286 (1) | -0.50766 | 0.24107 (1) | -0.22361 | 0.60714 (1) |
| *Snowella*_0TU37S04 | -0.35714 | 0.38938 (1) | 0.66667 | 0.07624 (1) | 0.63474 | 0.10089 (1) | 0.34157 | 0.41012 (1) |
| *Aphanizomenon*_NIES81 | 0.2338 | 0.60714 (1) | 0.76376 | 0.031429 (1) | 0.73699 | 0.048286 (1) | 0.75068 | 0.043571 (1) |
| *Cyanobium*_PCC-6307 | 0 | 1 (1) | -0.02381 | 0.97679 (1) | -0.15569 | 0.71567 (1) | -0.58554 | 0.13869 (1) |
| *Pseudanabaena*_PCC-7429 | -0.27545 | 0.50526 (1) | 0.74253 | 0.041865 (1) | 0.75301 | 0.037847 (1) | 0.49088 | 0.21979 (1) |
| *Dolichospermum*_NIES41 | -0.19279 | 0.65 (1) | 0.6145 | 0.1129 (1) | 0.60607 | 0.11875 (1) | 0.64202 | 0.096131 (1) |
| *Cuspidothrix*_LMECYA_163 | 0.37126 | 0.3627 (1) | 0.37126 | 0.3627 (1) | 0.48795 | 0.2189 (1) | 0.76087 | 0.038839 (1) |

*r*: Spearman’s coefficient; significant values (*p* < 0.05) after Bonferroni correction were highlighted in red. *p-*uncorrected values are shown outside of the parenthesis, while Bonferroni corrected values are shown inside the parenthesis.

**Table S9**. Number of total reads of 16S rRNA classified to bacteria (genus level) without cyanobacteria.

| **Taxa** | **TL** | | | | | **RA** | | | | | **SU** | | | | | **SI** | | | | |
| --- | --- | --- | --- | --- | --- | --- | --- | --- | --- | --- | --- | --- | --- | --- | --- | --- | --- | --- | --- | --- |
|  | 1 | 2 | 3 | Average | % | 1 | 2 | 3 | Average | % | 1 | 2 | 3 | Average | % | 1 | 2 | 3 | Average | % |
| *Rheinheimera* | 73 | 88 | 78 | 79.7 | 0.1 | 271 | 250 | 291 | 270.7 | 0.4 | 6169 | 5625 | 6265 | 6019.7 | 6.0 | 35026 | 19936 | 28779 | 27913.7 | 16.8 |
| *Roseomonas* | 4726 | 6237 | 3893 | 4952.0 | 3.7 | 8754 | 8662 | 9565 | 8993.7 | 11.7 | 1480 | 1195 | 1752 | 1475.7 | 1.5 | 1510 | 1304 | 1142 | 1318.7 | 0.8 |
| Burkholderiaceae | 8612 | 10994 | 8766 | 9457.3 | 7.1 | 1409 | 1033 | 860 | 1100.7 | 1.4 | 3439 | 2893 | 4106 | 3479.3 | 3.5 | 468 | 332 | 304 | 368.0 | 0.2 |
| *Rhodoferax* | 124 | 140 | 104 | 122.7 | 0.1 | 2463 | 2552 | 2574 | 2529.7 | 3.3 | 1785 | 1446 | 1819 | 1683.3 | 1.7 | 5451 | 4255 | 4149 | 4618.3 | 2.8 |
| *Paucibacter* | 9 | 0 | 0 | 3.0 | 0.0 | 14 | 25 | 36 | 25.0 | 0.0 | 3079 | 3003 | 3411 | 3164.3 | 3.1 | 4314 | 2949 | 2834 | 3365.7 | 2.0 |
| Rhodanobacteraceae | 4398 | 5334 | 4333 | 4688.3 | 3.5 | 164 | 160 | 177 | 167.0 | 0.2 | 126 | 131 | 192 | 149.7 | 0.1 | 0 | 0 | 0 | 0.0 | 0.0 |
| *Aeromonas* | 35 | 74 | 55 | 54.7 | 0.0 | 70 | 49 | 68 | 62.3 | 0.1 | 1294 | 1077 | 1518 | 1296.3 | 1.3 | 1294 | 1231 | 2148 | 1557.7 | 0.9 |
| *Undibacterium* | 8 | 18 | 27 | 17.7 | 0.0 | 58 | 61 | 70 | 63.0 | 0.1 | 360 | 361 | 385 | 368.7 | 0.4 | 3103 | 1793 | 2012 | 2302.7 | 1.4 |
| *Flavobacterium* | 5445 | 6802 | 6334 | 6193.7 | 4.7 | 4442 | 3068 | 2171 | 3227.0 | 4.2 | 29332 | 28973 | 30947 | 29750.7 | 29.5 | 83098 | 50842 | 58051 | 63997.0 | 38.4 |
| env.OPS_17 | 5563 | 7196 | 7081 | 6613.3 | 5.0 | 391 | 287 | 237 | 305.0 | 0.4 | 2605 | 2501 | 2740 | 2615.3 | 2.6 | 630 | 310 | 454 | 464.7 | 0.3 |
| Chitinophagaceae | 3541 | 4338 | 3091 | 3656.7 | 2.8 | 430 | 307 | 241 | 326.0 | 0.4 | 1793 | 1935 | 2203 | 1977.0 | 2.0 | 2408 | 986 | 1661 | 1685.0 | 1.0 |
| Microscillaceae | 4185 | 5686 | 5941 | 5270.7 | 4.0 | 1175 | 1187 | 1184 | 1182.0 | 1.5 | 1280 | 1058 | 1433 | 1257.0 | 1.2 | 235 | 177 | 251 | 221.0 | 0.1 |
| NS11-12_marine_group | 2545 | 3345 | 2824 | 2904.7 | 2.2 | 651 | 517 | 431 | 533.0 | 0.7 | 2221 | 2549 | 2604 | 2458.0 | 2.4 | 1939 | 715 | 1173 | 1275.7 | 0.8 |
| Saprospiraceae | 3685 | 5396 | 4137 | 4406.0 | 3.3 | 844 | 550 | 506 | 633.3 | 0.8 | 938 | 904 | 1027 | 956.3 | 0.9 | 217 | 165 | 146 | 176.0 | 0.1 |
| *Emticicia* | 10 | 25 | 32 | 22.3 | 0.0 | 25 | 12 | 13 | 16.7 | 0.0 | 3 | 0 | 5 | 2.7 | 0.0 | 3867 | 4122 | 2009 | 3332.7 | 2.0 |
| *Fluviicola* | 1447 | 2063 | 1749 | 1753.0 | 1.3 | 348 | 217 | 170 | 245.0 | 0.3 | 1217 | 1268 | 1258 | 1247.7 | 1.2 | 506 | 290 | 373 | 389.7 | 0.2 |
| *Sediminibacterium* | 372 | 730 | 448 | 516.7 | 0.4 | 973 | 833 | 715 | 840.3 | 1.1 | 590 | 651 | 723 | 654.7 | 0.7 | 1448 | 872 | 1027 | 1115.7 | 0.7 |
| OPB56 | 1700 | 2425 | 1880 | 2001.7 | 1.5 | 1035 | 752 | 628 | 805.0 | 1.0 | 481 | 448 | 549 | 492.7 | 0.5 | 327 | 175 | 316 | 272.7 | 0.2 |
| *Dinghuibacter* | 1623 | 2309 | 1526 | 1819.3 | 1.4 | 529 | 316 | 244 | 363.0 | 0.5 | 473 | 517 | 559 | 516.3 | 0.5 | 126 | 80 | 90 | 98.7 | 0.1 |
| Methylacidiphilaceae | 18 | 34 | 63 | 38.3 | 0.0 | 2927 | 2250 | 1883 | 2353.3 | 3.1 | 7 | 0 | 30 | 12.3 | 0.0 | 4790 | 2193 | 3401 | 3461.3 | 2.1 |
| Verrucomicrobiae | 3226 | 3807 | 3768 | 3600.3 | 2.7 | 606 | 404 | 340 | 450.0 | 0.6 | 202 | 157 | 222 | 193.7 | 0.2 | 110 | 25 | 68 | 67.7 | 0.0 |
| Pedosphaeraceae | 1702 | 2654 | 2249 | 2201.7 | 1.7 | 1804 | 1361 | 1159 | 1441.3 | 1.9 | 214 | 299 | 301 | 271.3 | 0.3 | 226 | 152 | 125 | 167.7 | 0.1 |
| *Prosthecobacter* | 1271 | 1993 | 1091 | 1451.7 | 1.1 | 903 | 658 | 580 | 713.7 | 0.9 | 166 | 165 | 229 | 186.7 | 0.2 | 0 | 40 | 18 | 19.3 | 0.0 |
| LD29 | 7 | 14 | 0 | 7.0 | 0.0 | 467 | 360 | 289 | 372.0 | 0.5 | 434 | 311 | 428 | 391.0 | 0.4 | 687 | 700 | 471 | 619.3 | 0.4 |
| Verrucomicrobiaceae | 775 | 1074 | 516 | 788.3 | 0.6 | 68 | 60 | 29 | 52.3 | 0.1 | 358 | 256 | 379 | 331.0 | 0.3 | 0 | 0 | 0 | 0.0 | 0.0 |
| DEV007 | 653 | 1088 | 490 | 743.7 | 0.6 | 44 | 53 | 44 | 47.0 | 0.1 | 20 | 16 | 17 | 17.7 | 0.0 | 9 | 0 | 0 | 3.0 | 0.0 |
| *Luteolibacter* | 371 | 557 | 143 | 357.0 | 0.3 | 32 | 18 | 17 | 22.3 | 0.0 | 420 | 352 | 412 | 394.7 | 0.4 | 0 | 3 | 0 | 1.0 | 0.0 |
| IMCC26134 | 338 | 397 | 405 | 380.0 | 0.3 | 113 | 109 | 82 | 101.3 | 0.1 | 24 | 23 | 31 | 26.0 | 0.0 | 56 | 32 | 45 | 44.3 | 0.0 |
| hgcI_clade | 4036 | 5007 | 6046 | 5029.7 | 3.8 | 404 | 336 | 245 | 328.3 | 0.4 | 908 | 895 | 1009 | 937.3 | 0.9 | 24 | 10 | 11 | 15.0 | 0.0 |
| CL500-29_marine_group | 2824 | 3866 | 3728 | 3472.7 | 2.6 | 632 | 442 | 410 | 494.7 | 0.6 | 678 | 540 | 641 | 619.7 | 0.6 | 1173 | 1232 | 884 | 1096.3 | 0.7 |
| PeM15 | 206 | 294 | 368 | 289.3 | 0.2 | 648 | 607 | 586 | 613.7 | 0.8 | 143 | 125 | 150 | 139.3 | 0.1 | 1073 | 964 | 772 | 936.3 | 0.6 |
| *Mycobacterium* | 745 | 1192 | 757 | 898.0 | 0.7 | 233 | 213 | 252 | 232.7 | 0.3 | 542 | 379 | 522 | 481.0 | 0.5 | 42 | 51 | 19 | 37.3 | 0.0 |
| 67-14 | 103 | 109 | 158 | 123.3 | 0.1 | 134 | 117 | 123 | 124.7 | 0.2 | 143 | 95 | 125 | 121.0 | 0.1 | 898 | 854 | 477 | 743.0 | 0.4 |
| Microtrichales | 863 | 1145 | 1093 | 1033.7 | 0.8 | 154 | 171 | 120 | 148.3 | 0.2 | 93 | 78 | 86 | 85.7 | 0.1 | 37 | 23 | 18 | 26.0 | 0.0 |
| IMCC26207 | 60 | 88 | 86 | 78.0 | 0.1 | 135 | 122 | 118 | 125.0 | 0.2 | 34 | 35 | 38 | 35.7 | 0.0 | 621 | 694 | 373 | 562.7 | 0.3 |
| *Candidatus*_*Planktophila* | 655 | 822 | 800 | 759.0 | 0.6 | 45 | 22 | 14 | 27.0 | 0.0 | 173 | 195 | 229 | 199.0 | 0.2 | 0 | 0 | 0 | 0.0 | 0.0 |
| *Conexibacter* | 17 | 35 | 26 | 26.0 | 0.0 | 64 | 51 | 68 | 61.0 | 0.1 | 6 | 0 | 5 | 3.7 | 0.0 | 447 | 409 | 183 | 346.3 | 0.2 |
| *Candidatus*_*Limnoluna* | 374 | 466 | 325 | 388.3 | 0.3 | 10 | 0 | 10 | 6.7 | 0.0 | 123 | 105 | 118 | 115.3 | 0.1 | 0 | 0 | 0 | 0.0 | 0.0 |
| *Gaiellales* | 157 | 190 | 210 | 185.7 | 0.1 | 63 | 69 | 62 | 64.7 | 0.1 | 6 | 6 | 14 | 8.7 | 0.0 | 227 | 184 | 138 | 183.0 | 0.1 |
| Caldilineaceae | 12 | 34 | 20 | 22.0 | 0.0 | 10361 | 9803 | 10116 | 10093.3 | 13.1 | 10 | 0 | 7 | 5.7 | 0.0 | 6021 | 4411 | 3664 | 4698.7 | 2.8 |
| Roseiflexaceae | 1973 | 2782 | 2356 | 2370.3 | 1.8 | 349 | 225 | 166 | 246.7 | 0.3 | 122 | 104 | 149 | 125.0 | 0.1 | 796 | 404 | 517 | 572.3 | 0.3 |
| *Candidatus_Chloroploca* | 0 | 0 | 0 | 0.0 | 0.0 | 773 | 695 | 690 | 719.3 | 0.9 | 0 | 0 | 0 | 0.0 | 0.0 | 970 | 357 | 604 | 643.7 | 0.4 |
| JG30-KF-CM45 | 362 | 602 | 341 | 435.0 | 0.3 | 0 | 6 | 7 | 4.3 | 0.0 | 6 | 0 | 0 | 2.0 | 0.0 | 718 | 646 | 278 | 547.3 | 0.3 |
| A4b | 786 | 1111 | 1239 | 1045.3 | 0.8 | 5 | 0 | 0 | 1.7 | 0.0 | 0 | 0 | 0 | 0.0 | 0.0 | 2 | 0 | 0 | 0.7 | 0.0 |
| Anaerolineaceae | 229 | 394 | 373 | 332.0 | 0.3 | 14 | 5 | 10 | 9.7 | 0.0 | 0 | 0 | 2 | 0.7 | 0.0 | 42 | 17 | 33 | 30.7 | 0.0 |
| KD4-96 | 80 | 117 | 116 | 104.3 | 0.1 | 2 | 14 | 0 | 5.3 | 0.0 | 14 | 12 | 25 | 17.0 | 0.0 | 55 | 43 | 33 | 43.7 | 0.0 |
| RBG-13-54-9 | 15 | 27 | 25 | 22.3 | 0.0 | 15 | 10 | 8 | 11.0 | 0.0 | 19 | 12 | 13 | 14.7 | 0.0 | 123 | 103 | 85 | 103.7 | 0.1 |
| OLB15 | 0 | 0 | 0 | 0.0 | 0.0 | 33 | 25 | 19 | 25.7 | 0.0 | 0 | 0 | 0 | 0.0 | 0.0 | 91 | 68 | 79 | 79.3 | 0.0 |
| Anaerolineae | 97 | 120 | 125 | 114.0 | 0.1 | 0 | 0 | 0 | 0.0 | 0.0 | 0 | 0 | 0 | 0.0 | 0.0 | 0 | 6 | 0 | 2.0 | 0.0 |
| others | 43756 | 61477 | 49780 | 51671.0 | 39.0 | 40475 | 34266 | 34085 | 36275.3 | 47.2 | 35376 | 32878 | 40950 | 36401.3 | 36.1 | 47412 | 34816 | 28561 | 36929.7 | 22.2 |
| **sum** | 113812 | 154696 | 128996 | 132501.3 | 100.0 | 85559 | 73310 | 71713 | 76860.7 | 100.0 | 98906 | 93573 | 109628 | 100702.3 | 100.0 | 212617 | 138971 | 147776 | 166454.7 | 100.0 |

Replicates (1, 2 and 3). TL: The Lake in Central Park – New York, USA; RA: Raczyńskie Lake, Poland; SU: Sulejów Reservoir, Poland; SP: Singapore urban reservoir.

**Table S10**. Diversity indices for taxonomic units (16S rRNA amplicons).

| **Index** | **TL** | | | | | **RA** | | | | | **SU** | | | | | **SP** | | | | |
| --- | --- | --- | --- | --- | --- | --- | --- | --- | --- | --- | --- | --- | --- | --- | --- | --- | --- | --- | --- | --- |
|  | 1 | 2 | 3 | Avg | Stdev | 1 | 2 | 3 | Avg | Stdev | 1 | 2 | 3 | Avg | Stdev | 1 | 2 | 3 | Avg | Stdev |
| Dominance_D | 0.024 | 0.023 | 0.024 | 0.024 | 0.0007244 | 0.040 | 0.048 | 0.055 | 0.048 | 0.005940 | 0.104 | 0.113 | 0.097 | 0.104 | 0.0062824 | 0.211 | 0.182 | 0.215 | 0.203 | 0.014957 |
| Shannon_H | 4.40 | 4.45 | 4.42 | 4.43 | 0.0222261 | 4.25 | 4.14 | 4.06 | 4.15 | 0.075834 | 3.68 | 3.62 | 3.73 | 3.68 | 0.0466262 | 2.78 | 2.95 | 2.70 | 2.81 | 0.106035 |
| Equitability_J | 0.72 | 0.72 | 0.72 | 0.72 | 0.0001886 | 0.72 | 0.70 | 0.69 | 0.71 | 0.011726 | 0.64 | 0.63 | 0.64 | 0.64 | 0.0040434 | 0.48 | 0.51 | 0.47 | 0.49 | 0.017986 |
| ACE | 445.8 | 478.0 | 457.0 | 460.2 | 13.346993 | 362.2 | 357.2 | 353.2 | 357.5 | 3.681787 | 328.2 | 310.4 | 339.0 | 325.8 | 11.791899 | 344.0 | 314.2 | 293.5 | 317.2 | 20.72781 |

Replicates (1, 2 and 3); Avg: Average; Stdev: standard deviation. TL: The Lake in Central Park – New York, USA; RA: Raczyńskie Lake, Poland; SU: Sulejów Reservoir, Poland; SP: Singapore urban reservoir.

**Table S11**. Kruskal Wallis and Dunn’s post hoc tests to evaluate differences between diversity indices

| Shannon-Wiener (H) | | | | |  | Richness (ACE) | | | | |
| --- | --- | --- | --- | --- | --- | --- | --- | --- | --- | --- |
| **Kruskal-Wallis test for equal medians** | | | |  |  | **Kruskal-Wallis test for equal medians** | | | |  |
|  |  |  |  |  |  |  |  |  |  |  |
| H (chi2): | 10.38 |  |  |  |  | H (chi2): | 9.359 |  |  |  |
| Hc (tie corrected): | 10.38 |  |  |  |  | Hc (tie corrected): | 9.359 |  |  |  |
| p (same): | 0.01556 |  |  |  |  | p (same): | 0.02488 |  |  |  |
| There is a significant difference between sample medians | | | | |  | There is a significant difference between sample medians | | | | |
| **Dunns post hoc test (*p* values):** | | |  |  |  | **Dunns post hoc test (*p* values):** | | |  |  |
|  | TL | RA | SU | SP |  |  | TL | RA | SU | SP |
| TL |  | 1 | 0.2492 | 0.01341 |  | TL |  | 1 | 0.07643 | 0.04525 |
| RA |  |  | 1 | 0.2492 |  | RA |  |  | 1 | 0.6775 |
| SU |  |  |  | 1 |  | SU |  |  |  | 1 |
|  |  |  |  |  |  |  |  |  |  |  |
| Eveness (J) | | | | |  | Simpson's dominance (D) | | | | |
| **Kruskal-Wallis test for equal medians** | | | |  |  | **Kruskal-Wallis test for equal medians** | | | |  |
|  |  |  |  |  |  |  |  |  |  |  |
| H (chi2): | 10.38 |  |  |  |  | H (chi2): | 10.38 |  |  |  |
| Hc (tie corrected): | 10.42 |  |  |  |  | Hc (tie corrected): | 10.38 |  |  |  |
| p (same): | 0.01531 |  |  |  |  | p (same): | 0.01556 |  |  |  |
| There is a significant difference between sample medians | | | | |  | There is a significant difference between sample medians | | | | |
| **Dunns post hoc test (*p* values):** | | |  |  |  | **Dunns post hoc test (*p* values):** | | |  |  |
|  | TL | RA | SU | SP |  |  | TL | RA | SU | SP |
| TL |  | 1 | 0.2492 | 0.01341 |  | TL |  | 1 | 0.2492 | 0.01341 |
| RA |  |  | 1 | 0.2492 |  | RA |  |  | 1 | 0.2492 |
| SU |  |  |  | 1 |  | SU |  |  |  | 1 |

Significant values (*p* < 0.05) highlighted in red. The Bonferroni correction of *p*-values was included for the Dunn’s post hoc test.

**Table S12**. Scores and loadings for the construction of PCA – bacterioplankton communities.

| Scores | | |  | Loadings | | |
| --- | --- | --- | --- | --- | --- | --- |
| **Samples** | **PC 1** | **PC 2** |  | **Important taxa** | **PC 1** | **PC 2** |
| TL1 | -20.799 | -9.3866 |  | *Microcystis*_PCC-7914 | -0.53211 | -0.34993 |
| TL2 | -15.762 | -6.018 |  | *Planktothrix*_NIVA-CYA_15 | 0.039355 | 0.44232 |
| TL3 | -18.791 | -7.986 |  | *Aphanizomenon*_MDT14a | 0.68852 | -0.61671 |
| RA1 | -18.101 | -1.8232 |  | *Snowella*_0TU37S04 | -0.09529 | 0.056485 |
| RA2 | -18.174 | -1.8393 |  | *Aphanizomenon*_NIES81 | -0.23226 | -0.04429 |
| RA3 | -19.037 | -2.1527 |  | *Cyanobium*_PCC-6307 | -0.00133 | 0.058381 |
| SU1 | 27.508 | -16.488 |  | *Pseudanabaena*_PCC-7429 | -0.02877 | -0.00482 |
| SU2 | 27.202 | -15.291 |  | *Dolichospermum*_NIES41 | -0.00888 | -0.01865 |
| SU3 | 26.724 | -16.093 |  | *Cuspidothrix*_LMECYA_163 | -0.0059 | -0.01993 |
| SP1 | 9.5623 | 25.105 |  | *Rheinheimera* | 0.12934 | 0.269 |
| SP2 | 8.9302 | 25.518 |  | *Roseomonas* | -0.06178 | -0.02473 |
| SP3 | 10.737 | 26.455 |  | Burkholderiaceae | -0.02809 | -0.06046 |
|  |  |  |  | *Rhodoferax* | 0.012972 | 0.036218 |
|  |  |  |  | *Paucibacter* | 0.037592 | 0.012414 |
|  |  |  |  | *Flavobacterium* | 0.39004 | 0.45599 |
|  |  |  |  | env.OPS_17 | -0.01477 | -0.04112 |
|  |  |  |  | *Emticicia* | 0.008737 | 0.03799 |
|  |  |  |  | Methylacidiphilaceae | -0.00379 | 0.036105 |
|  |  |  |  | Caldilineaceae | -0.04388 | 0.042258 |

TL: The Lake in Central Park – New York, USA; RA: Raczyńskie Lake, Poland; SU: Sulejów Reservoir, Poland; SP: Singapore urban reservoir.

**Table S13**. Kruskal Wallis and Dunn’s post hoc test to evaluate differences between PCA samples utilizing the PC1 scores.

| **Kruskal-Wallis test for equal medians** | | | |  |
| --- | --- | --- | --- | --- |
|  |  |  |  |  |
| H (chi2): | 9.359 |  |  |  |
| Hc (tie corrected): | 9.359 |  |  |  |
| p (same): | 0.02488 |  |  |  |
|  |  |  |  |  |
| There is a significant difference between sample medians | | | | |
| **Dunn's post hoc test:** | |  |  |  |
|  |  |  |  |  |
|  | TL | RA | SU | SP |
| TL |  | 1 | 0.0092 | 0.0119 |
| RA |  |  | 0.0113 | 0.0141 |
| SU |  |  |  | 0.3082 |

Significant values (p < 0.05) highlighted in red. The Bonferroni correction of *p*-values was included for the Dunn’s post hoc test.

**Table S14**. Spearman’s correlations between cyanobacteria strains and their attached consortia (other bacteria).

| **Bacterial taxa** | *Microcystis*_PCC-7914 | | *Planktothrix*_NIVA-CYA_15 | | *Aphanizomenon*_MDT14a | | *Aphanizomenon*_NIES81 | | *Snowella*_0TU37S04 | | *Cyanobium*_PCC-6307 | | *Pseudanabaena*_PCC-7429 | | *Dolichospermum*_NIES41 | | *Cuspidothrix*_LMECYA_163 | |
| --- | --- | --- | --- | --- | --- | --- | --- | --- | --- | --- | --- | --- | --- | --- | --- | --- | --- | --- |
|  | *r* | *p-corr.* | *r* | *p-corr.* | *r* | *p-corr.* | *r* | *p-corr.* | *r* | *p-corr.* | *r* | *p-corr.* | *r* | *p-corr.* | *r* | *p-corr.* | *r* | *p-corr.* |
| Rheinheimera | -0.66 | 9.9E-01 | 0.61 | 1.0E+00 | -0.27 | 1.0E+00 | -0.39 | 1.0E+00 | -0.26 | 1.0E+00 | 0.32 | 1.0E+00 | -0.39 | 1.0E+00 | -0.49 | 1.0E+00 | -0.71 | 5.6E-01 |
| Roseomonas | **0.75** | **5.0E-02** | -0.35 | 1.0E+00 | -0.35 | 1.0E+00 | **0.88** | **2.1E-02** | 0.72 | 3.7E-01 | -0.34 | 1.0E+00 | **0.86** | **2.3E-02** | **0.75** | **5.0E-02** | **0.78** | **4.2E-02** |
| Burkholderiaceae | **0.75** | **5.0E-02** | -0.66 | 9.9E-01 | 0.07 | 1.0E+00 | -0.28 | 1.0E+00 | -0.49 | 1.0E+00 | -0.05 | 1.0E+00 | -0.31 | 1.0E+00 | -0.26 | 1.0E+00 | 0.12 | 1.0E+00 |
| Rhodoferax | **-0.76** | **4.8E-02** | **0.88** | **2.1E-02** | -0.29 | 1.0E+00 | -0.06 | 1.0E+00 | 0.21 | 1.0E+00 | 0.44 | 1.0E+00 | -0.03 | 1.0E+00 | -0.16 | 1.0E+00 | -0.50 | 1.0E+00 |
| Paucibacter | **-0.78** | **4.2E-02** | 0.42 | 1.0E+00 | 0.54 | 1.0E+00 | -0.51 | 1.0E+00 | -0.22 | 1.0E+00 | 0.35 | 1.0E+00 | -0.46 | 1.0E+00 | -0.31 | 1.0E+00 | -0.38 | 1.0E+00 |
| Rhodanobacteraceae | **0.79** | **4.1E-02** | -0.48 | 1.0E+00 | -0.23 | 1.0E+00 | -0.22 | 1.0E+00 | -0.42 | 1.0E+00 | 0.11 | 1.0E+00 | -0.27 | 1.0E+00 | -0.33 | 1.0E+00 | 0.00 | 1.0E+00 |
| Aeromonas | **-0.91** | **2.1E-02** | 0.52 | 1.0E+00 | 0.29 | 1.0E+00 | -0.57 | 1.0E+00 | -0.38 | 1.0E+00 | 0.24 | 1.0E+00 | -0.54 | 1.0E+00 | -0.46 | 1.0E+00 | -0.62 | 1.0E+00 |
| Undibacterium | **-0.75** | **5.0E-02** | **0.92** | **1.4E-02** | -0.26 | 1.0E+00 | -0.42 | 1.0E+00 | -0.09 | 1.0E+00 | **0.75** | **5.0E-02** | -0.39 | 1.0E+00 | -0.53 | 1.0E+00 | **-0.77** | **4.0E-02** |
| Flavobacterium | **-0.83** | **3.5E-02** | **0.80** | **3.7E-02** | 0.00 | 1.0E+00 | -0.55 | 1.0E+00 | -0.22 | 1.0E+00 | 0.65 | 1.0E+00 | -0.51 | 1.0E+00 | -0.55 | 1.0E+00 | **-0.75** | **5.0E-02** |
| env.OPS_17 | 0.67 | 9.7E-01 | -0.63 | 1.0E+00 | 0.11 | 1.0E+00 | -0.35 | 1.0E+00 | -0.55 | 1.0E+00 | -0.03 | 1.0E+00 | -0.39 | 1.0E+00 | -0.33 | 1.0E+00 | 0.06 | 1.0E+00 |
| Chitinophagaceae | 0.24 | 1.0E+00 | -0.22 | 1.0E+00 | 0.00 | 1.0E+00 | **-0.75** | **5.0E-02** | **-0.80** | **3.7E-02** | 0.33 | 1.0E+00 | **-0.76** | **4.8E-02** | **-0.75** | **5.0E-02** | -0.47 | 1.0E+00 |
| Microscillaceae | **0.84** | **2.7E-02** | -0.62 | 1.0E+00 | -0.09 | 1.0E+00 | -0.11 | 1.0E+00 | -0.33 | 1.0E+00 | -0.07 | 1.0E+00 | -0.15 | 1.0E+00 | -0.16 | 1.0E+00 | 0.20 | 1.0E+00 |
| NS11-12_marine_group | 0.36 | 1.0E+00 | -0.54 | 1.0E+00 | 0.45 | 1.0E+00 | -0.50 | 1.0E+00 | -0.58 | 1.0E+00 | 0.04 | 1.0E+00 | -0.50 | 1.0E+00 | -0.34 | 1.0E+00 | 0.02 | 1.0E+00 |
| Saprospiraceae | **0.79** | **4.2E-02** | -0.58 | 1.0E+00 | -0.09 | 1.0E+00 | -0.19 | 1.0E+00 | -0.40 | 1.0E+00 | 0.02 | 1.0E+00 | -0.23 | 1.0E+00 | -0.23 | 1.0E+00 | 0.12 | 1.0E+00 |
| Emticicia | -0.67 | 8.3E-01 | **0.92** | **1.4E-02** | -0.39 | 1.0E+00 | -0.38 | 1.0E+00 | -0.09 | 1.0E+00 | **0.75** | **5.0E-02** | -0.36 | 1.0E+00 | -0.54 | 1.0E+00 | **-0.78** | **4.2E-02** |
| Fluviicola | 0.40 | 1.0E+00 | -0.64 | 1.0E+00 | 0.32 | 1.0E+00 | -0.55 | 1.0E+00 | **-0.75** | **5.0E-02** | -0.09 | 1.0E+00 | -0.58 | 1.0E+00 | -0.44 | 1.0E+00 | -0.07 | 1.0E+00 |
| Sediminibacterium | -0.35 | 1.0E+00 | 0.69 | 7.5E-01 | -0.19 | 1.0E+00 | 0.17 | 1.0E+00 | 0.48 | 1.0E+00 | 0.56 | 1.0E+00 | 0.21 | 1.0E+00 | 0.10 | 1.0E+00 | -0.10 | 1.0E+00 |
| OPB56 | **0.90** | **1.5E-02** | -0.58 | 1.0E+00 | -0.18 | 1.0E+00 | 0.05 | 1.0E+00 | -0.17 | 1.0E+00 | -0.04 | 1.0E+00 | 0.01 | 1.0E+00 | -0.03 | 1.0E+00 | 0.28 | 1.0E+00 |
| Dinghuibacter | **0.79** | **4.2E-02** | -0.62 | 1.0E+00 | -0.04 | 1.0E+00 | -0.16 | 1.0E+00 | -0.38 | 1.0E+00 | -0.01 | 1.0E+00 | -0.19 | 1.0E+00 | -0.18 | 1.0E+00 | 0.17 | 1.0E+00 |
| Methylacidiphilaceae | -0.39 | 1.0E+00 | **0.86** | **2.3E-02** | -0.54 | 1.0E+00 | 0.26 | 1.0E+00 | 0.54 | 1.0E+00 | 0.53 | 1.0E+00 | 0.28 | 1.0E+00 | 0.06 | 1.0E+00 | -0.28 | 1.0E+00 |
| Verrucomicrobiae | **0.83** | **3.5E-02** | -0.50 | 1.0E+00 | -0.24 | 1.0E+00 | -0.15 | 1.0E+00 | -0.35 | 1.0E+00 | 0.06 | 1.0E+00 | -0.19 | 1.0E+00 | -0.26 | 1.0E+00 | 0.06 | 1.0E+00 |
| Pedosphaeraceae | **0.96** | **5.2E-03** | -0.53 | 1.0E+00 | -0.33 | 1.0E+00 | 0.35 | 1.0E+00 | 0.12 | 1.0E+00 | -0.13 | 1.0E+00 | 0.31 | 1.0E+00 | 0.22 | 1.0E+00 | 0.46 | 1.0E+00 |
| Prosthecobacter | **0.91** | **2.1E-02** | -0.57 | 1.0E+00 | -0.25 | 1.0E+00 | 0.21 | 1.0E+00 | -0.03 | 1.0E+00 | -0.07 | 1.0E+00 | 0.17 | 1.0E+00 | 0.11 | 1.0E+00 | 0.39 | 1.0E+00 |
| LD29 | **-0.85** | **2.8E-02** | 0.73 | 9.5E-02 | -0.07 | 1.0E+00 | -0.11 | 1.0E+00 | 0.13 | 1.0E+00 | 0.28 | 1.0E+00 | -0.08 | 1.0E+00 | -0.12 | 1.0E+00 | -0.45 | 1.0E+00 |
| Verrucomicrobiaceae | 0.66 | 9.9E-01 | -0.66 | 9.9E-01 | 0.16 | 1.0E+00 | -0.28 | 1.0E+00 | -0.49 | 1.0E+00 | -0.03 | 1.0E+00 | -0.31 | 1.0E+00 | -0.23 | 1.0E+00 | 0.14 | 1.0E+00 |
| DEV007 | 0.75 | 5.0E-02 | -0.45 | 1.0E+00 | -0.23 | 1.0E+00 | -0.19 | 1.0E+00 | -0.37 | 1.0E+00 | 0.17 | 1.0E+00 | -0.22 | 1.0E+00 | -0.29 | 1.0E+00 | 0.04 | 1.0E+00 |
| Luteolibacter | 0.33 | 1.0E+00 | **-0.76** | **4.8E-02** | 0.61 | 1.0E+00 | -0.34 | 1.0E+00 | -0.51 | 1.0E+00 | -0.22 | 1.0E+00 | -0.34 | 1.0E+00 | -0.10 | 1.0E+00 | 0.25 | 1.0E+00 |
| IMCC26134 | **0.88** | **2.1E-02** | -0.46 | 1.0E+00 | -0.32 | 1.0E+00 | -0.05 | 1.0E+00 | -0.23 | 1.0E+00 | 0.07 | 1.0E+00 | -0.09 | 1.0E+00 | -0.18 | 1.0E+00 | 0.12 | 1.0E+00 |
| hgcI_clade | **0.77** | **4.0E-02** | -0.57 | 1.0E+00 | -0.08 | 1.0E+00 | -0.24 | 1.0E+00 | -0.44 | 1.0E+00 | -0.02 | 1.0E+00 | -0.28 | 1.0E+00 | -0.28 | 1.0E+00 | 0.06 | 1.0E+00 |
| CL500-29_marine_group | **0.69** | **7.5E-01** | -0.32 | 1.0E+00 | -0.25 | 1.0E+00 | -0.31 | 1.0E+00 | -0.43 | 1.0E+00 | 0.28 | 1.0E+00 | -0.34 | 1.0E+00 | -0.42 | 1.0E+00 | -0.11 | 1.0E+00 |
| PeM15 | -0.38 | 1.0E+00 | **0.91** | **2.1E-02** | -0.66 | 9.9E-01 | 0.13 | 1.0E+00 | 0.40 | 1.0E+00 | 0.64 | 1.0E+00 | 0.14 | 1.0E+00 | -0.13 | 1.0E+00 | -0.43 | 1.0E+00 |
| Mycobacterium | **0.75** | **5.0E-02** | -0.75 | 5.0E-02 | 0.22 | 1.0E+00 | -0.15 | 1.0E+00 | -0.37 | 1.0E+00 | -0.16 | 1.0E+00 | -0.17 | 1.0E+00 | -0.07 | 1.0E+00 | 0.32 | 1.0E+00 |
| 67-14 | -0.55 | 1.0E+00 | **0.91** | **2.1E-02** | -0.32 | 1.0E+00 | -0.32 | 1.0E+00 | 0.04 | 1.0E+00 | **0.84** | **2.7E-02** | -0.28 | 1.0E+00 | -0.45 | 1.0E+00 | -0.64 | 1.0E+00 |
| Microtrichales | **0.83** | **3.5E-02** | -0.52 | 1.0E+00 | -0.22 | 1.0E+00 | -0.14 | 1.0E+00 | -0.34 | 1.0E+00 | 0.05 | 1.0E+00 | -0.18 | 1.0E+00 | -0.24 | 1.0E+00 | 0.09 | 1.0E+00 |
| IMCC26207 | -0.49 | 1.0E+00 | **0.94** | **9.2E-03** | -0.42 | 1.0E+00 | -0.20 | 1.0E+00 | 0.14 | 1.0E+00 | **0.83** | **3.5E-02** | -0.17 | 1.0E+00 | -0.37 | 1.0E+00 | -0.59 | 1.0E+00 |
| Candidatus_Planktophila | **0.76** | **4.8E-02** | -0.61 | 1.0E+00 | 0.00 | 1.0E+00 | -0.29 | 1.0E+00 | -0.50 | 1.0E+00 | -0.02 | 1.0E+00 | -0.33 | 1.0E+00 | -0.30 | 1.0E+00 | 0.07 | 1.0E+00 |
| Conexibacter | -0.44 | 1.0E+00 | **0.90** | **2.6E-02** | -0.38 | 1.0E+00 | -0.17 | 1.0E+00 | 0.19 | 1.0E+00 | **0.85** | **2.8E-02** | -0.13 | 1.0E+00 | -0.32 | 1.0E+00 | -0.52 | 1.0E+00 |
| Candidatus_Limnoluna | 0.70 | 6.4E-01 | -0.62 | 1.0E+00 | 0.05 | 1.0E+00 | -0.31 | 1.0E+00 | -0.52 | 1.0E+00 | 0.01 | 1.0E+00 | -0.35 | 1.0E+00 | -0.31 | 1.0E+00 | 0.07 | 1.0E+00 |
| Gaiellales | 0.32 | 1.0E+00 | 0.41 | 1.0E+00 | -0.63 | 1.0E+00 | -0.25 | 1.0E+00 | -0.11 | 1.0E+00 | **0.78** | **4.4E-02** | -0.26 | 1.0E+00 | -0.52 | 1.0E+00 | -0.44 | 1.0E+00 |
| Caldilineaceae | 0.15 | 1.0E+00 | 0.31 | 1.0E+00 | -0.42 | 1.0E+00 | **0.89** | **2.3E-02** | **0.98** | **1.5E-04** | -0.03 | 1.0E+00 | **0.90** | **2.6E-02** | **0.75** | **5.0E-02** | 0.51 | 1.0E+00 |
| Roseiflexaceae | 0.68 | 7.4E-01 | -0.26 | 1.0E+00 | -0.38 | 1.0E+00 | -0.31 | 1.0E+00 | -0.43 | 1.0E+00 | 0.31 | 1.0E+00 | -0.35 | 1.0E+00 | -0.47 | 1.0E+00 | -0.19 | 1.0E+00 |
| Candidatus_Chloroploca | -0.06 | 1.0E+00 | 0.59 | 1.0E+00 | -0.45 | 1.0E+00 | 0.62 | 1.0E+00 | **0.85** | **2.8E-02** | 0.30 | 1.0E+00 | 0.65 | 1.0E+00 | 0.45 | 1.0E+00 | 0.19 | 1.0E+00 |
| JG30-KF-CM45 | 0.06 | 1.0E+00 | 0.51 | 1.0E+00 | -0.45 | 1.0E+00 | -0.45 | 1.0E+00 | -0.24 | 1.0E+00 | **0.93** | **1.3E-02** | -0.44 | 1.0E+00 | -0.64 | 1.0E+00 | -0.57 | 1.0E+00 |
| A4b | **0.76** | **4.8E-02** | -0.44 | 1.0E+00 | -0.24 | 1.0E+00 | -0.24 | 1.0E+00 | -0.43 | 1.0E+00 | 0.10 | 1.0E+00 | -0.28 | 1.0E+00 | -0.35 | 1.0E+00 | -0.03 | 1.0E+00 |
| Anaerolineaceae | 0.72 | 3.7E-01 | -0.36 | 1.0E+00 | -0.30 | 1.0E+00 | -0.27 | 1.0E+00 | -0.43 | 1.0E+00 | 0.19 | 1.0E+00 | -0.31 | 1.0E+00 | -0.40 | 1.0E+00 | -0.09 | 1.0E+00 |
| KD4-96 | 0.36 | 1.0E+00 | -0.04 | 1.0E+00 | -0.39 | 1.0E+00 | -0.56 | 1.0E+00 | -0.62 | 1.0E+00 | 0.43 | 1.0E+00 | -0.59 | 1.0E+00 | -0.73 | 1.0E+00 | -0.51 | 1.0E+00 |
| RBG-13-54-9 | -0.47 | 1.0E+00 | **0.87** | **2.4E-02** | -0.31 | 1.0E+00 | -0.36 | 1.0E+00 | -0.01 | 1.0E+00 | **0.89** | **1.9E-02** | -0.33 | 1.0E+00 | -0.49 | 1.0E+00 | -0.64 | 1.0E+00 |
| OLB15 | -0.43 | 1.0E+00 | **0.88** | **2.1E-02** | -0.41 | 1.0E+00 | 0.02 | 1.0E+00 | 0.35 | 1.0E+00 | 0.70 | 6.4E-01 | 0.06 | 1.0E+00 | -0.14 | 1.0E+00 | -0.40 | 1.0E+00 |
| Anaerolineae | **0.76** | **4.8E-02** | -0.44 | 1.0E+00 | -0.26 | 1.0E+00 | -0.26 | 1.0E+00 | -0.44 | 1.0E+00 | 0.12 | 1.0E+00 | -0.30 | 1.0E+00 | -0.37 | 1.0E+00 | -0.05 | 1.0E+00 |

*r*: Spearman’s coefficient; significant r values (p < 0.05) are bold; *p*-corr.: *p*-values after Bonferroni correction; significant values are highlighted in red.

**Table S15**. Composition of nutrient-transforming genes in metagenomic shotguns.

|  | **Genes** | **TL_1** | | **TL_2** | | **RA_1** | | **RA_2** | | **SU_1** | | **SU_2** | | **SI_1** | | **SI_2** | |
| --- | --- | --- | --- | --- | --- | --- | --- | --- | --- | --- | --- | --- | --- | --- | --- | --- | --- |
|  |  | HN | Normalized | HN | Normalized | HN | Normalized | HN | Normalized | HN | Normalized | HN | Normalized | HN | Normalized | HN | Normalized |
| N-cycling genes | *ure*B | 1923 | 1.07E-01 | 1340 | 9.52E-02 | 1527 | 7.83E-02 | 3273 | 9.12E-02 | 1226 | 7.93E-02 | 1795 | 7.83E-02 | 445 | 2.09E-02 | 657 | 2.47E-02 |
|  | *gln*A | 834 | 4.63E-02 | 600 | 4.26E-02 | 547 | 2.80E-02 | 1339 | 3.73E-02 | 478 | 3.09E-02 | 749 | 3.27E-02 | 218 | 1.02E-02 | 315 | 1.19E-02 |
|  | *gcv*T | 2 | 1.11E-04 | 8 | 5.68E-04 | 3 | 1.54E-04 | 15 | 4.18E-04 | 30 | 1.94E-03 | 38 | 1.66E-03 | 35 | 1.64E-03 | 108 | 4.07E-03 |
|  | *glt*B | 1 | 5.55E-05 | 0 | 0.00E+00 | 0 | 0.00E+00 | 1 | 2.79E-05 | 0 | 0.00E+00 | 0 | 0.00E+00 | 0 | 0.00E+00 | 0 | 0.00E+00 |
|  | *nif*K | 1 | 5.55E-05 | 3 | 2.13E-04 | 1045 | 5.36E-02 | 2253 | 6.28E-02 | 1887 | 1.22E-01 | 3406 | 1.49E-01 | 193 | 9.06E-03 | 156 | 5.87E-03 |
|  | *nif*B | 0 | 0.00E+00 | 0 | 0.00E+00 | 0 | 0.00E+00 | 8 | 2.23E-04 | 0 | 0.00E+00 | 1 | 4.36E-05 | 0 | 0.00E+00 | 0 | 0.00E+00 |
|  | *nar*B | 532 | 2.95E-02 | 395 | 2.81E-02 | 216 | 1.11E-02 | 535 | 1.49E-02 | 271 | 1.75E-02 | 280 | 1.22E-02 | 87 | 4.08E-03 | 139 | 5.23E-03 |
|  | *nar*Z | 37 | 2.05E-03 | 29 | 2.06E-03 | 113 | 5.79E-03 | 404 | 1.13E-02 | 284 | 1.84E-02 | 438 | 1.91E-02 | 456 | 2.14E-02 | 855 | 3.22E-02 |
|  | *nar*H | 10 | 5.55E-04 | 25 | 1.78E-03 | 33 | 1.69E-03 | 181 | 5.04E-03 | 93 | 6.02E-03 | 188 | 8.20E-03 | 171 | 8.03E-03 | 329 | 1.24E-02 |
|  | *nar*G | 3 | 1.66E-04 | 1 | 7.10E-05 | 9 | 4.61E-04 | 42 | 1.17E-03 | 16 | 1.04E-03 | 19 | 8.29E-04 | 24 | 1.13E-03 | 51 | 1.92E-03 |
|  | *nap*A | 0 | 0.00E+00 | 0 | 0.00E+00 | 0 | 0.00E+00 | 0 | 0.00E+00 | 6 | 3.88E-04 | 0 | 0.00E+00 | 1 | 4.70E-05 | 0 | 0.00E+00 |
|  | *nor*B | 2 | 1.11E-04 | 12 | 8.52E-04 | 3 | 1.54E-04 | 17 | 4.74E-04 | 6 | 3.88E-04 | 22 | 9.59E-04 | 18 | 8.45E-04 | 42 | 1.58E-03 |
|  | *nir*K | 3 | 1.66E-04 | 0 | 0.00E+00 | 0 | 0.00E+00 | 1 | 2.79E-05 | 4 | 2.59E-04 | 8 | 3.49E-04 | 0 | 0.00E+00 | 0 | 0.00E+00 |
|  | *nos*Z | 0 | 0.00E+00 | 0 | 0.00E+00 | 2 | 1.03E-04 | 0 | 0.00E+00 | 0 | 0.00E+00 | 1 | 4.36E-05 | 3 | 1.41E-04 | 0 | 0.00E+00 |
|  | *nxr*A | 0 | 0.00E+00 | 0 | 0.00E+00 | 1 | 5.13E-05 | 6 | 1.67E-04 | 0 | 0.00E+00 | 1 | 4.36E-05 | 1 | 4.70E-05 | 7 | 2.64E-04 |
|  | *nxr*B | 1 | 5.55E-05 | 1 | 7.10E-05 | 0 | 0.00E+00 | 1 | 2.79E-05 | 1 | 6.47E-05 | 2 | 8.72E-05 | 1 | 4.70E-05 | 4 | 1.51E-04 |
|  | *amo*A | 0 | 0.00E+00 | 0 | 0.00E+00 | 0 | 0.00E+00 | 0 | 0.00E+00 | 0 | 0.00E+00 | 0 | 0.00E+00 | 1 | 4.70E-05 | 0 | 0.00E+00 |
|  | _c_*amo*A | 0 | 0.00E+00 | 1 | 7.10E-05 | 0 | 0.00E+00 | 0 | 0.00E+00 | 0 | 0.00E+00 | 0 | 0.00E+00 | 0 | 0.00E+00 | 6 | 2.26E-04 |
|  |  |  |  |  |  |  |  |  |  |  |  |  |  |  |  |  |  |
| P-cycling genes | *pho*B | 46 | 2.55E-03 | 96 | 6.82E-03 | 52 | 2.67E-03 | 225 | 6.27E-03 | 30 | 1.94E-03 | 78 | 3.40E-03 | 9 | 4.23E-04 | 28 | 1.05E-03 |
|  | *pho*R | 26 | 1.44E-03 | 42 | 2.98E-03 | 26 | 1.33E-03 | 78 | 2.17E-03 | 48 | 3.11E-03 | 76 | 3.31E-03 | 24 | 1.13E-03 | 36 | 1.36E-03 |
|  | *pho*L | 3 | 1.66E-04 | 17 | 1.21E-03 | 4 | 2.05E-04 | 8 | 2.23E-04 | 10 | 6.47E-04 | 19 | 8.29E-04 | 8 | 3.76E-04 | 13 | 4.90E-04 |
|  | *pho*P | 5 | 2.77E-04 | 14 | 9.94E-04 | 3 | 1.54E-04 | 13 | 3.62E-04 | 3 | 1.94E-04 | 8 | 3.49E-04 | 3 | 1.41E-04 | 2 | 7.53E-05 |
|  | *pho*H | 0 | 0.00E+00 | 1 | 7.10E-05 | 6 | 3.08E-04 | 17 | 4.74E-04 | 0 | 0.00E+00 | 1 | 4.36E-05 | 1 | 4.70E-05 | 10 | 3.77E-04 |
|  | *ppk*1 | 87 | 4.83E-03 | 53 | 3.76E-03 | 68 | 3.49E-03 | 197 | 5.49E-03 | 36 | 2.33E-03 | 92 | 4.01E-03 | 19 | 8.92E-04 | 77 | 2.90E-03 |
|  | *ppx* | 0 | 0.00E+00 | 2 | 1.42E-04 | 0 | 0.00E+00 | 8 | 2.23E-04 | 12 | 7.76E-04 | 33 | 1.44E-03 | 0 | 0.00E+00 | 9 | 3.39E-04 |
|  | 16S rRNA | 18023 | 1 | 14081 | 1 | 19505 | 1 | 35896 | 1 | 15458 | 1 | 22931 | 1 | 21298 | 1 | 26555 | 1 |

HN: hit numbers; normalized to the total number of 16S rRN gene. TL: The Lake in Central Park – New York, USA; RA: Raczyńskie Lake, Poland; SU: Sulejów Reservoir, Poland; SP: Singapore urban reservoir.

**Table S16**. Scores and correlations for the construction of PCA – nutrient cycling genes.

|  | Scores | | |  | Correlations to axis (coordinates) | | |
| --- | --- | --- | --- | --- | --- | --- | --- |
|  | **Samples** | **PC 1** | **PC 2** |  | **genes** | **PC 1** | **PC 2** |
| **N-cycling genes** | TL1 | -0.54326 | 2.0378 |  | *ure*B | -0.90547 | 0.0432 |
|  | TL2 | -0.1719 | 0.55516 |  | *gln*A | 0.16549 | 0.19068 |
|  | RA1 | -0.44033 | -0.4905 |  | *gcv*T | 0.9903 | -0.035633 |
|  | RA2 | -0.42786 | -0.041535 |  | *glt*B | -0.26773 | 0.81971 |
|  | SU1 | 0.28665 | -1.2285 |  | *nif*B | -0.20231 | -0.03749 |
|  | SU2 | 2.374 | 0.29851 |  | *nif*K | -0.25094 | -0.52941 |
|  | SP1 | -0.54755 | -0.73749 |  | *nap*A | -0.12693 | -0.56386 |
|  | SP2 | -0.52972 | -0.39337 |  | *nar*G | 0.97307 | -0.098657 |
|  |  |  |  |  | *nar*B | 0.011057 | 0.91952 |
|  |  |  |  |  | *nar*Z | 0.96543 | 0.0475 |
|  |  |  |  |  | *nar*H | 0.89543 | 0.0297 |
|  |  |  |  |  | *nir*K | -0.43932 | 0.49965 |
|  |  |  |  |  | *nor*B | 0.99258 | -0.037581 |
|  |  |  |  |  | *nos*Z | 0.082072 | -0.54031 |
|  |  |  |  |  | *amo*A | 0.11582 | -0.4964 |
|  |  |  |  |  | _c_*amo*A | 0.9613 | 0.14405 |
|  |  |  |  |  | *nxr*A | 0.96885 | 0.047088 |
|  |  |  |  |  | *nxr*B | 0.97873 | 0.15651 |
|  |  |  |  |  |  |  |  |
|  | Scores | | |  | Correlations to axis (coordinates) | | |
|  | **Samples** | **PC 1** | **PC 2** |  | **genes** | **PC 1** | **PC 2** |
| **P-cycling genes** | TL1 | -0.24597 | 0.78505 |  | *pho*H | 0.80441 | -0.48076 |
|  | TL2 | 1.5662 | 2.7554 |  | *pho*B | 0.5652 | 0.71039 |
|  | RA1 | -2.2751 | -0.56144 |  | *pho*L | 0.88927 | 0.044236 |
|  | RA2 | -0.2101 | 0.38969 |  | *pho*P | 0.44306 | 0.87697 |
|  | SU1 | -0.45922 | 0.06784 |  | *pho*R | 0.95897 | 0.01453 |
|  | SU2 | 4.0478 | -1.7911 |  | *ppk*1 | 0.83289 | -0.082483 |
|  | SP1 | -1.7057 | -0.79163 |  | *ppx* | 0.63082 | -0.61492 |
|  | SP2 | -0.71803 | -0.85377 |  |  |  |  |

TL: The Lake in Central Park – New York, USA; RA: Raczyńskie Lake, Poland; SU: Sulejów Reservoir, Poland; SP: Singapore urban reservoir.

**Acknowledgements**

Nutrient and field parameter data from TL are available from the USGS Water Data for the Nation: National Water Information database (U.S. Geological Survey, 2025). Any use of trade, firm, or product names is for descriptive purposes only and does not imply endorsement by the U.S. Government. Environmental data concerning RA and SU water bodies are not publicly available; they are stored in an internal database at the UNESCO Chair in Ecohydrology and Applied Ecology at the University of Lodz, Poland. The environmental monitoring data for SP are agency in-house records and are not publicly available. Access may be requested from the agency subject to data sharing policies.

**Reference**s

Callahan, B. J., McMurdie, P. J., Rosen, M. J., Han, A. W., Johnson, A. J. A., & Holmes, S. P. 2016. DADA2: High-resolution sample inference from Illumina amplicon data.
Nature Methods, 13(7), 581–583. <https://doi.org/10.1038/nmeth.3869>

Harper, D.A.T. (ed.). 1999. Numerical Palaeobiology. John Wiley & Sons

R core Team 2023. R: A language and environment for statistical computing. R foundation for Statistical Computing, Vienna, Austria. <https://www.R-project.org/>

Te S.H., Gin K.Y-H., 2011. The dynamics of cyanobacteria and microcystin production in a tropical reservoir of Singapore. Harmful Algae, 10, 319-329, https://doi.org/10.1016/j.hal.2010.11.006

U.S. Geological Survey. 2025. USGS Water Data for the Nation: National Water Information System database. U.S. Geological Survey. <https://doi.org/10.5066/F7P55KJN> (accessed August 22, 2025)
